# Epithelial *Atg5* Deficiency Intensifies Caspase‐11 Activation, Fueling Extracellular mtDNA Release to Activate cGAS–STING–NLRP3 Axis in Macrophages During *Pseudomonas* Infection

**DOI:** 10.1002/mco2.70239

**Published:** 2025-06-15

**Authors:** Junyi Wang, Lei Zhang, Yingying Liu, Yao Liu, Anying Xiong, Qin Ran, Xiang He, Vincent Kam Wai Wong, Colin Combs, Guoping Li, Min Wu

**Affiliations:** ^1^ Laboratory of Allergy and Precision Medicine, Department of Respiratory Medicine, Chengdu Institute of Respiratory Health Affiliated Hospital of Southwest Jiaotong University, the Third People's Hospital of Chengdu Chengdu China; ^2^ State Key Laboratory of Quality Research in Chinese Medicine Macau University of Science & Technology Taipa Macao China; ^3^ Department of Biomedical Sciences, School of Medicine and Health Sciences University of North Dakota Grand Forks North Dakota USA; ^4^ Wenzhou Institute University of Chinese Academy of Sciences Wenzhou Zhejiang China

**Keywords:** ATG5, caspase‐11, cGAS/STING, epithelial cells, mitochondrial DNA, *Pseudomonas aeruginosa*

## Abstract

*Pseudomonas aeruginosa* (*P. aeruginosa*) infections pose a significant threat to public health, underscoring the need for deeper insights into host cellular defenses. This study explores the critical role of autophagy‐related protein 5 (ATG5) in lung epithelial cells during *P. aeruginosa* infection. Single‐cell RNA transcriptomics revealed a pronounced enrichment of autophagy pathways in type II alveolar epithelial cells (AEC2). Using a conditional *Atg5* knockout murine model, we demonstrated that ATG5 deficiency in AEC2 compromises survival, hampers bacterial clearance, and increases pathogen dissemination. Additionally, the loss of ATG5 exacerbated inflammatory responses, notably through the activation of the AKT/PI3K/NF‐κB axis and pyroptosis, which culminated in severe lung injury and epithelial barrier disruption. Mechanistically, the absence of ATG5 disrupted mitophagy, leading to intensified mitochondrial damage. This exacerbated condition coupled with the activation of gasdermin D (GSDMD) by the noncanonical caspase‐11, enhancing the release of mitochondrial DNA (mtDNA), which in turn activated cGAS–STING–NLRP3 signaling in macrophages. These findings highlight the essential role of ATG5 in modulating immune responses and suggest potential therapeutic targets for managing *P. aeruginosa*‐induced pulmonary infections.

## Graphical abstract

This work illuminates the essential role of epithelial ATG5 in defending against *P. aeruginosa*. *Atg5* deficiency impairs bacterial clearance and aggravates inflammation via AKT/PI3K/NF‐κB, inflammasome activation, and pyroptosis. Mechanistically, impaired mitophagy intensifies mitochondrial damage. This, combined with the enhanced activation of GSDMD mediated by the noncanonical caspase‐11 inflammasome, amplifies the extracellular release of mtDNA, triggering cGAS–STING–NLRP3 signaling in macrophages and amplifying lung injury.

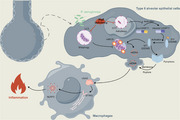

## 1 Introduction

*Pseudomonas aeruginosa*, an opportunistic Gram‐negative bacterium, is notably prevalent in infections afflicting immunocompromised individuals, those with severe burns, or patients suffering from chronic pulmonary conditions such as cystic fibrosis and chronic obstructive pulmonary disease [1]. This pathogen is part of the ESKAPE group, which includes *Enterococcus faecium*, *Staphylococcus aureus*, *Klebsiella pneumoniae*, *Acinetobacter baumannii*, *P. aeruginosa*, and *Enterobacter*. These pathogens are known for their multidrug resistance, positioning them as critical targets for novel therapeutic strategies [2]. The World Health Organization has prioritized carbapenem‐resistant strains of *P. aeruginosa* for new drug development, underlining the urgent need for innovative interventions in clinical settings [3]. Therefore, a deeper understanding of host–pathogen interactions is crucial for developing strategies that leverage host defensive processes to prevent and treat bacterial infections effectively.

Autophagy, a fundamental cellular process, plays a crucial role in maintaining cellular integrity and energy homeostasis during stress conditions by degrading and recycling cytoplasmic components. This process involves the formation of autophagosomes, double‐membraned vesicles that encapsulate and transport cellular debris to lysosomes. The initiation of canonical autophagy involves the fusion of FIP200‐ and autophagy‐related protein (ATG) 16L1‐positive vesicles, leading to the formation of a prophagophore. Two interconnected ubiquitin‐like enzymatic cascades, involving ATG7, ATG3, and a complex comprising ATG12, ATG5, and ATG16L1, lipidate the mammalian paralogue of the ATG8 family, LC3‐I, transforming it into LC3‐II. The production of LC3‐II is essential for the assembly, elongation, and closure of autophagosomes, illustrating the intricate molecular choreography of this process [4].

Pyroptosis, a form of programmed cell death, plays pivotal roles in cellular immune defense through its classical and nonclassical pathways. The classical pathway, triggered by caspase‐1 activation via inflammasome complexes like NLRP3, detects microbial products or cellular stress, leading to the cleavage of gasdermin D (GSDMD). This cleavage results in pore formation in the cell membrane, cell lysis, and the release of proinflammatory cytokines, such as IL‐1β and IL‐18. In contrast, the nonclassical pathway involves caspase‐11 (caspase‐4/5 in humans), which also can cleave GSDMD to induce pyroptosis [5]. Concurrently, the cyclic GMP–AMP synthase (cGAS)–stimulator of interferon genes (STING pathway, activated by cytosolic DNA from pathogens or damaged mitochondria, triggers the production of type I interferons, enhancing antiviral and antibacterial defenses.

Recent studies have highlighted the pivotal role of autophagy in defending mammals against microbial threats. Notably, the depletion of key autophagic proteins, like ATG5, ATG16L1, or ATG7 in macrophages, not only enhances the growth of *Mycobacterium tuberculosis* but also increases host susceptibility [6]. The dynamic interplay between autophagy, inflammasomes, pyroptosis, and the cGAS‐STING pathway is essential for orchestrating cellular responses to infection. This is particularly crucial in the lungs, a primary site of attack by pathogens like *P. aeruginosa*, which can trigger severe immune reactions. Previous research from our group demonstrated increased inflammasome activation and pyroptosis in *P. aeruginosa*‐induced sepsis following *Atg7* depletion in mice [7], raising significant questions about how autophagy modulates the frontline defenses in lung epithelial cells. This study delves into the critical role of ATG5 within lung epithelial cells, orchestrating a multifaceted defense against *P. aeruginosa* infection. By regulating inflammatory responses, maintaining mitochondrial homeostasis, controlling caspase‐11 activation, and limiting the release of extracellular mitochondrial DNA (mtDNA), ATG5 enhances cellular resilience. These findings have deepened our understanding of the nuanced interplay between epithelial autophagy and host defense mechanisms, underscoring potential therapeutic strategies to modulate inflammation and enhance pathogen clearance in respiratory infections.

## 2 Results

### 2.1 Alveolar Epithelial Deficiency in *Atg5* Increased Susceptibility to *P. aeruginosa* Infection

**FIGURE 1:**
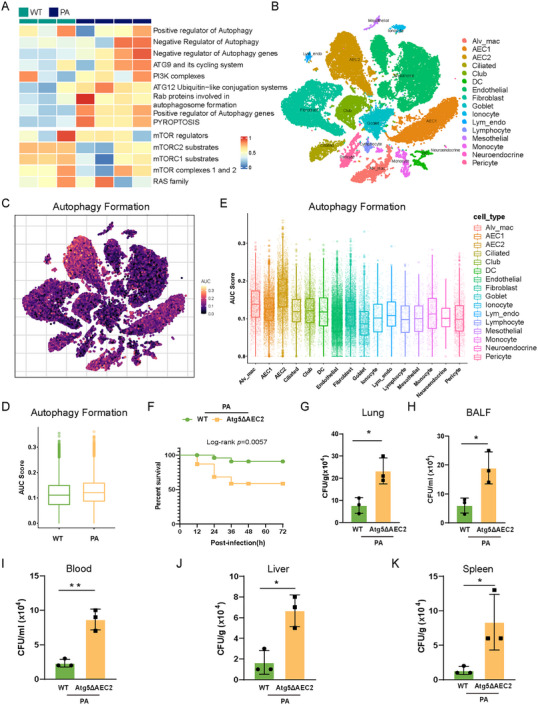
Alveolar epithelial deficiency in *Atg5* increased susceptibility to *P. aeruginosa* infection. (A) Heatmap showing enrichment for indicated gene signatures via ssGSEA. (B) t‐SNE visualization of 16 major cell types in lungs from *P. aeruginosa*‐infected and control mice. (C) t‐SNE map visualizing AUC scores of autophagy formation gene signature across all cells. (D, E) Box plots showing AUC scores of autophagy formation gene signature calculated across all cells within each group (D) or distributed across each cell type (E). (F) Survival rates are represented by Kaplan–Meier curves (*n* = 8). (G–K) Bacterial burden in the lung (G), BALF (H), blood (I), liver (J), and spleen (K) assessed by CFU assays at 24 h after infection. Data represent means ± SD of three independent experiments, *p < 0.05, **p < 0.01 by unpaired *t*‐test. AEC1, type I alveolar epithelial cells; AEC2, type II alveolar epithelial cells; Alv_mac, alveolar macrophages; Atg5ΔAEC2, type II alveolar epithelial cell‐specific *Atg5* conditional knockout; Ciliated, ciliated cells; Club, club cells; DC, dendritic cells; Endothelial, endothelial cells; Fibroblast, fibroblasts; Goblet, goblet cells; Ionocyte, ionocytes; Lym_endo, lymphatic endothelial cells; Lymphocyte, lymphocytes; Mesothelial, mesothelial cells; Monocyte, monocytes; Neuroendocrine, neuroendocrine cells; PA, *P. aeruginosa*; Pericyte, pericytes; WT, wildtype.

By analyzing our lung bulk RNA‐seq dataset, comprising three wild‐type (WT) mice and four *P. aeruginosa*‐infected mice at 24 h postinfection, we uncovered a pronounced enrichment of autophagy‐related pathways upon *P. aeruginosa* infection, as determined by single‐sample gene set enrichment analysis (ssGSEA; Figure 1A). To delve into the enrichment of autophagy formation signature across distinct cell types, we employed our single nucleus RNA sequencing (SnRNA‐seq) dataset derived from two WT mice and two *P. aeruginosa*‐infected mice at 24 h postinfection (Figures 1B and S1). AUCell analysis revealed an escalated autophagy formation signature enrichment in *P. aeruginosa*‐infected lungs compared to control lungs, with type II alveolar epithelial cells (AEC2) exhibiting the highest enrichment of this signature (Figure 1C–E). Through protein–protein interaction (PPI) analysis with the bulk RNA‐seq dataset (Figure S2), we found that ATG5, a pivotal protein involved in the autophagy formation, plays a key role in the interaction network within *P. aeruginosa*‐infected lungs. Therefore, we examined the role of ATG5 in AEC2 during *P. aeruginosa* infection, utilizing a mouse pneumonia model. PAO1 (1 × 10^7^ CFU/mouse) was intranasally delivered into each of AEC2‐specific *Atg5* conditional knockout (*Atg5* ^ΔAEC2^) mice and their littermate WT counterparts. Upon PAO1 infection, deficiency of *Atg5* in AEC2 reduced the survival rate, resulting in a mortality rate of 45% for *Atg5* ^ΔAEC2^ mice, while WT mice exhibited a mortality rate of 13% after 72 h (Figure 1F). Moreover, *Atg5* ^ΔAEC2^ mice exhibited impaired bacterial clearance in both lungs and bronchoalveolar lavage fluid (BALF) compared to WT mice (Figure 1G,H). Notably, there was a marked increase in bacterial burdens in the blood, spleen, and liver of *Atg5* ^ΔAEC2^ mice, indicating enhanced dissemination of *P. aeruginosa* (Figure 1I–K). Collectively, these findings underscore the indispensable role of ATG5 in epithelial cells for resistance to *P. aeruginosa* infection.

### 2.2 Alveolar Epithelial Deficiency in *Atg5* Exacerbated the Inflammatory Response and Activation of the AKT/PI3K/NF‐κB Axis in Lungs During *P. aeruginosa* Infection

**FIGURE 2:**
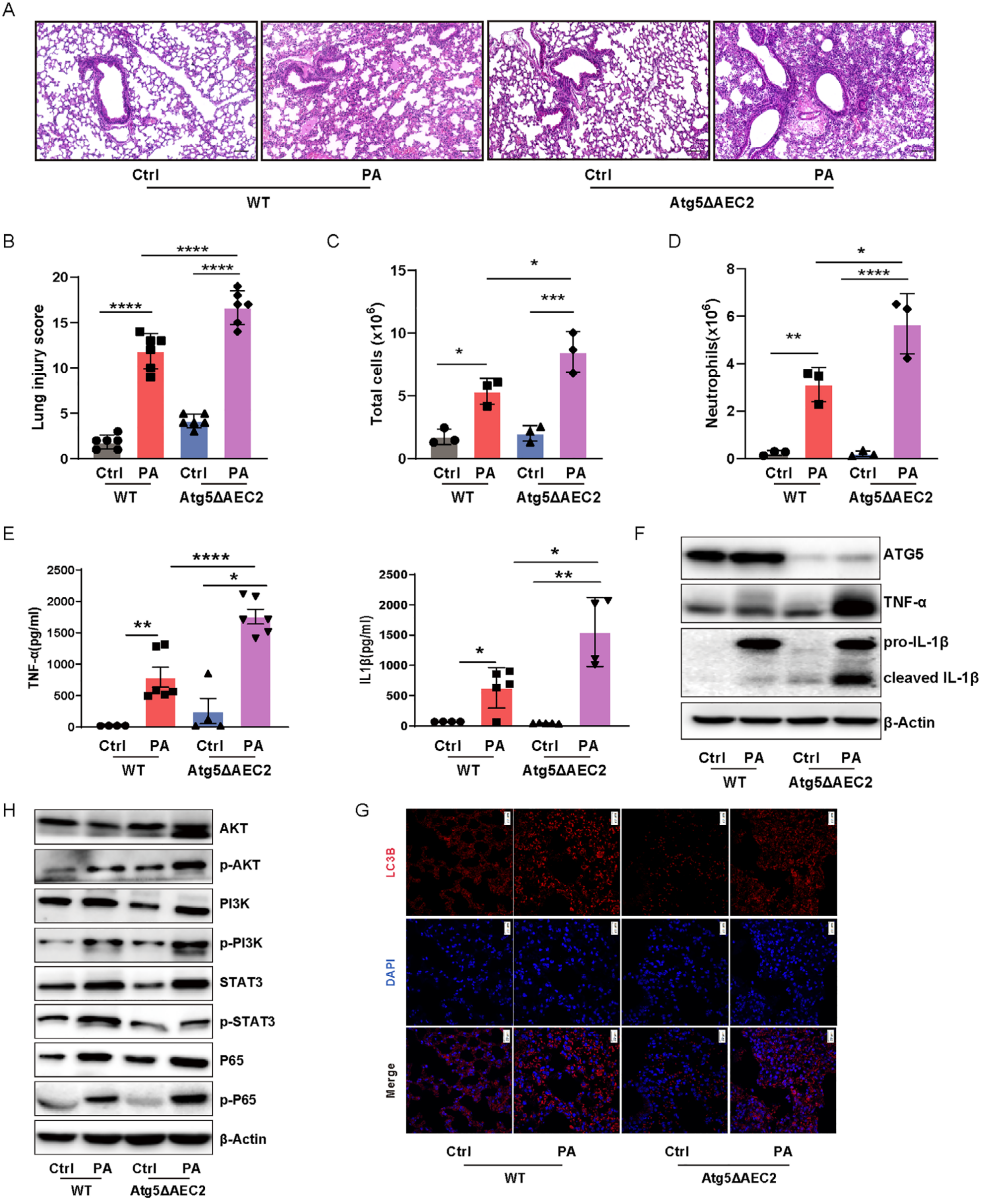
Alveolar epithelial deficiency in *Atg5* exacerbated the inflammatory response and activation of the AKT/PI3K/NF‐κB axis in lungs during *P. aeruginosa* infection. (A, B) Representative histological images and injury scores (B) of murine lungs via H&E staining (scale bar represents 10 µm). Data represent means ± SD (*n* = 6), ****p < 0.0001 by two‐way ANOVA with Bonferroni's post hoc test. (C, D) The number of total cells (C) and neutrophils (D) in BALF. Data represent means ± SD (*n* = 4), *p < 0.05, **p < 0.01, ***p < 0.001, ****p < 0.0001 by one‐way ANOVA with Bonferroni's post hoc test. (E) Secretion levels of TNF‐α (left) and IL‐1β (right) in BALF. Data represent means ± SD (*n* = 4–6), *p < 0.05, **p < 0.01, ****p < 0.0001 by two‐way ANOVA with Bonferroni's post hoc test. (F) The expression levels of ATG5, TNF‐α, and IL‐1β in mouse lungs determined by western blotting. (G) LC3B expression was analyzed by immunofluorescence in murine lungs (scale bar represents 20 µm). (H) The expression levels of AKT, PI3K, P65, STAT3, phosphorylated‐AKT, phosphorylated‐PI3K, phosphorylated‐P65, and phosphorylated‐STAT3 in mouse lungs determined by western blotting. Atg5ΔAEC2, type II alveolar epithelial cell‐specific Atg5 conditional knockout; Ctrl, control; PA, *P. aeruginosa*; WT, wildtype.

To gain deeper insight into the lethality associated with *P. aeruginosa* infection in *Atg5* ^ΔAEC2^ mice, lung samples were stained with hematoxylin and eosin staining (H&E) and scored in a blinded manner. The assessment revealed more severe lung injuries and heightened infiltration of inflammatory cells in *Atg5* ^ΔAEC2^ mice in comparison to their WT counterparts post *P. aeruginosa* infection (Figure 2A,B). Confirmation of increased total cells in BALF, notably neutrophils, further emphasized the heightened inflammatory state in *Atg5* ^ΔAEC2^ mice following exposure to PAO1 (Figure 2C,D). Given the pivotal role of cytokines in acute lung injury, we measured the levels of proinflammatory factors TNF‐α and IL‐1β, which were found to be significantly elevated in BALF from *Atg5* ^ΔAEC2^ mice compared to WT mice postinfection (Figure 2E). Western blotting validation underscored that the absence of *Atg5* and autophagy in AEC2 aggravated the production of TNF‐α and IL‐1β in the lungs during *P. aeruginosa* infection (Figures 2F,G and S3). Furthermore, immunoblotting revealed heightened activation of AKT, PI3K, and NF‐κB in the lungs of *Atg5* ^ΔAEC2^ mice compared to WT mice after PAO1 infection (Figures 2H and S3). Unexpectedly, while overall STAT3, another key proinflammatory pathway, increased, there was no evident elevation in phosphorylated‐STAT3 during *P. aeruginosa* infection in *Atg5* ^ΔAEC2^ mice (Figures 2H and S3). Similarly, our airway epithelial cell delivery experiments revealed that lungs treated with Atg5‐knockdown epithelial cells exhibited more severe lung injury and heightened infiltration of inflammatory cells following *P. aeruginosa* infection compared to those treated with control siRNA‐transfected epithelial cells. Notably, administration of the AKT inhibitor AKTi ½ or the NF‐κB inhibitor JSH‐23 significantly alleviated the exacerbated lung injury and inflammatory cell infiltration induced by epithelial Atg5 knockdown (Figure S3). These data suggest that epithelial ATG5 protects against lung injury upon infection, potentially by controlling the inflammatory response and regulating the AKT/PI3K/NF‐κB axis.

### 2.3 Loss of *Atg5* Intensified the Dysregulation of Tight Junctions in Alveolar Epithelial Cells During *P. aeruginosa* Infection

The lung epithelial barrier serves as a crucial defense against bacterial infiltration, preventing bacterial entry into the circulatory system and thereby mitigating bacterial dissemination [8]. To further understand bacterial dissemination in *Atg5* ^ΔAEC2^ mice, we conducted an assessment of the tight junction protein ZO‐1 through immunofluorescent staining (Figure S4A). Remarkably, ZO‐1 expression exhibited a marked decrease in lung samples of *Atg5* ^ΔAEC2^ mice compared to their WT counterparts in response to *P. aeruginosa* infection, a trend that was consistently confirmed by western blotting (Figure S4B,C). Correspondingly, the targeted knockdown of *Atg5* using siRNA resulted in a more pronounced reduction in ZO‐1 levels in the murine AEC2 (MLE‐12 cell line) following stimulation with *P. aeruginosa* (Figure S4D–F). These data underscore the critical role of ATG5 in the maintenance of epithelial tight junctions during *P. aeruginosa* infection

### 2.4 Alveolar Epithelial *Atg5* Deficiency Intensified Inflammasome Activation and Pyroptosis in Lungs Upon *P. aeruginosa* Infection

**FIGURE 3:**
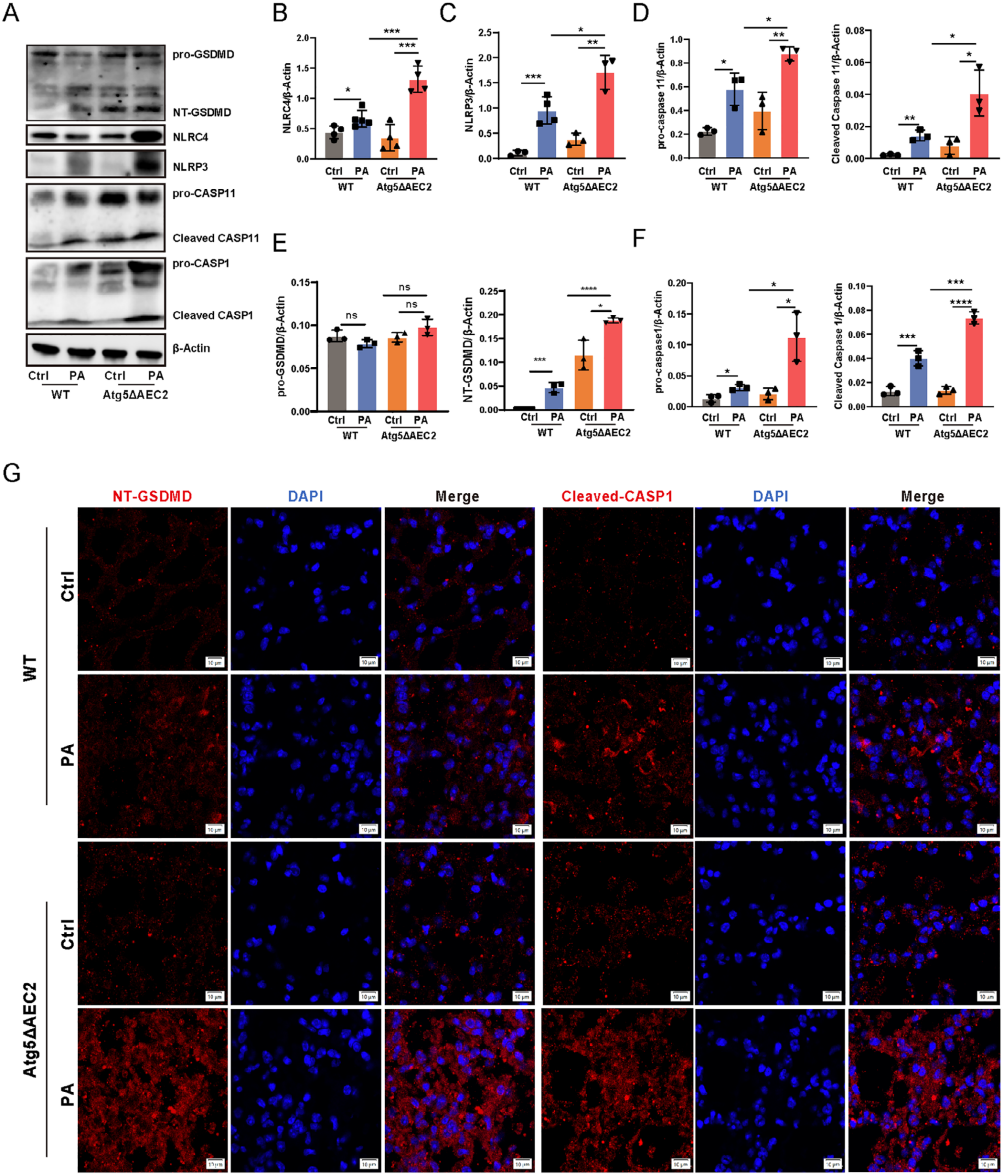
Alveolar epithelial *Atg5* deficiency intensified inflammasome activation and pyroptosis in lungs upon *P. aeruginosa* infection. (A) The expression levels of GSDMD, NLRP3, NLRC4, CASP11, and CASP1 in murine lungs determined by western blotting. (B–F) Semiquantitative analysis of (A). Data represent means ± SD of three to five independent experiments, *p < 0.05, **p < 0.01, ***p < 0.001 by two‐way ANOVA with Bonferroni's post hoc test. (G) The expression of NT‐GSDMD (left) and Cleaved CASP1 (right) was analyzed by immunofluorescence in mouse lungs (scale bar represents 5 µm). Atg5ΔAEC2, type II alveolar epithelial cell‐specific Atg5 conditional knockout; Ctrl, control; PA, *P. aeruginosa*; WT, wildtype.

Excessive inflammasome activation is known to contribute to exacerbated pathological damage [9]. We probed inflammasome expression levels through western blotting, revealing a substantial increase in NLRP3 and NLRC4 expression in *Atg5* ^ΔAEC2^ mice compared to WT mice upon *P. aeruginosa* infection (Figure 3A–F). Additionally, the levels of procaspase‐11 and cleaved caspase‐11 were elevated in mice lacking alveolar epithelial *Atg5* compared to control mice following infection (Figure 3A–F). These results underscore that *Atg5* deficiency intensifies the activation of pulmonary NLRP3, NLRC4, and caspase‐11 inflammasomes induced by *P. aeruginosa*. Given the established link between inflammasome activation and a form of inflammatory cell death known as pyroptosis [10], we investigated the expression of pyroptosis‐related proteins. Our analysis revealed an increase in procaspase‐1 and cleaved caspase‐1 in lung tissues of *Atg5* ^ΔAEC2^ mice after *P. aeruginosa* infection (Figure 3A–F). The activation of caspase‐1 results in the cleavage of gasdermin D (GSDMD), releasing the N‐terminal GSDMD (NT‐GSDMD) and inducing pyroptotic cell death [10]. Notably, the concurrent enhancement of NT‐GSDMD levels was observed alongside increased caspase‐1 cleavage in *Atg5* conditional knockout mice during infection (Figure 3A–F). Immunofluorescence further illustrated a substantial increase in cleaved caspase‐1 and NT‐GSDMD specks in *Atg5* ^ΔAEC2^ mice compared to WT mice (Figure 3G). Moreover, mice with AEC2 *Atg5* deletion exhibited an augmentation in cleaved IL‐1β compared to WT mice in response to *P. aeruginosa* challenge (Figure 2F). In summary, these findings establish the protective role of epithelial ATG5 against *P. aeruginosa*‐induced injury by finely modulating inflammasome activation and pyroptosis in the lungs.

### 2.5 Loss of *Atg5* Intensified Activation of Noncanonical Caspase‐11 Inflammasome and GSDMD in Alveolar Epithelial Cells Upon *P. aeruginosa* Infection

**FIGURE 4:**
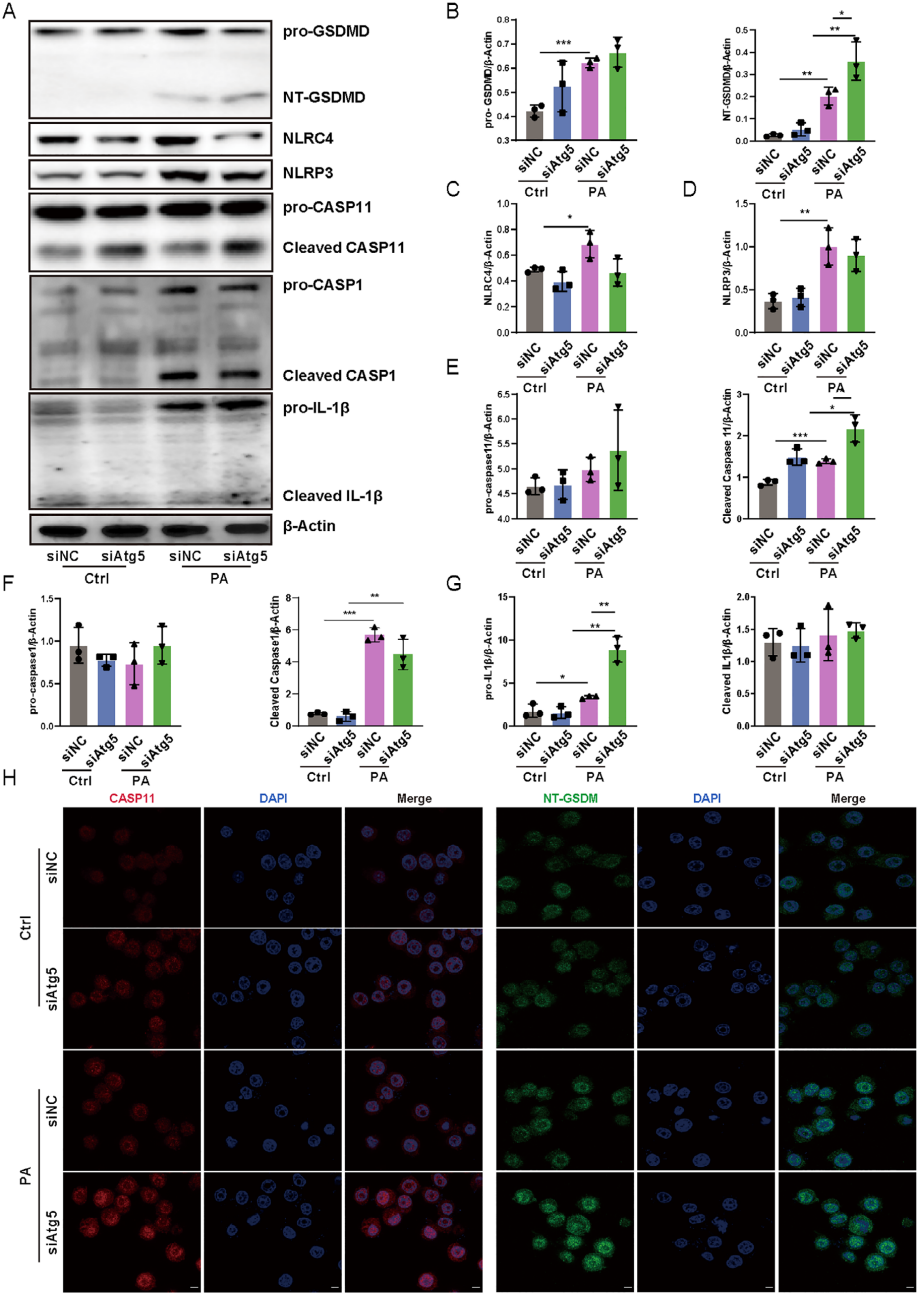
Loss of *Atg5* intensified activation of noncanonical caspase‐11 inflammasome and GSDMD in alveolar epithelial cells upon *P. aeruginosa* infection. (A) The expression levels of GSDMD, NLRP3, NLRC4, CASP11, CASP1, and IL‐1β in MLE‐12 cells determined by western blotting. (B–G) Semiquantitative analysis of (A). Data represent means ± SD of three independent experiments, *p < 0.05, **p < 0.01, ***p < 0.001 by two‐way ANOVA with Bonferroni's post hoc test. (H) The expression of CASP11 (left) and NT‐GSDMD (right) was analyzed by immunofluorescence in MLE‐12 cells (scale bar represents 5 µm). Ctrl, control; PA, *P. aeruginosa*; siAtg5, *Atg5* siRNA; siNC, control siRNA.

To further explore the mechanisms of *Atg5* in regulating inflammasomes and pyroptosis in alveolar epithelial cells during *P. aeruginosa* infection, we employed siRNA to interfere with *Atg5* expression in MLE‐12 cells. Unexpectedly, *Atg5* loss did not augment the activation of NLRP3 and NLRC4 in AEC2 following *P. aeruginosa* exposure (Figure 4A–G). Moreover, there was no increase observed in the levels of cleaved caspase‐1 and cleaved IL‐1β in *Atg5*‐silenced epithelial cells compared to control cells in response to *P. aeruginosa* infection (Figures 4A–G and S5). In contrast, an increase was noted in cleaved caspase‐11 in *Atg5* siRNA‐transfected cells compared to control siRNA‐transfected cells following exposure to *P. aeruginosa* (Figure 4A–G). While western blot showed unchanged procaspase‐11 levels, immunofluorescence demonstrated an increase in total caspase‐11 expression, possibly due to its ability to encompass both procaspase‐11 and its cleaved form (Figure 4H). Caspase‐11 orchestrates GSDMD activation through noncanonical inflammasome signaling. Consistently, this intensified activation of caspase‐11 coincided with an increase in NT‐GSDMD levels (Figure 4A–G). Immunofluorescence further illustrated augmented expression of NT‐GSDMD in cells transfected with *Atg5* siRNA compared to those transfected with control siRNA upon infection (Figure 4H). Taken together, these data indicate that ATG5 primarily implicates in the regulation of noncanonical caspase‐11 inflammasome‐mediated GSDMD activation in alveolar epithelial cells during *P. aeruginosa* infection.

### 2.6 Loss of *Atg5* Impedes Mitophagy and Aggravated Mitochondrial Damage in Epithelial Cells During *P. aeruginosa* Infection

**FIGURE 5:**
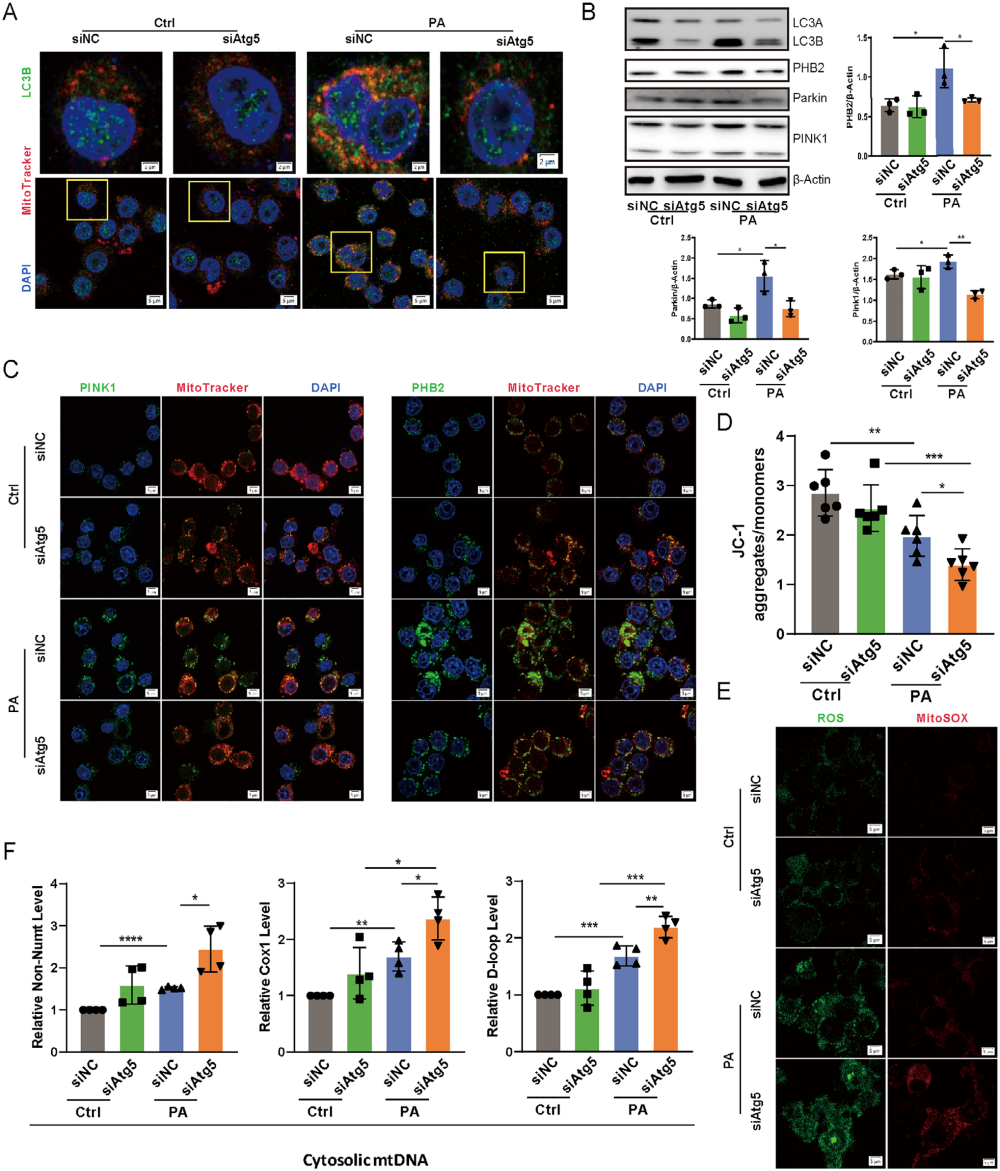
Loss of *Atg5* impedes mitophagy and aggravated mitochondrial damage in epithelial cells during *P. aeruginosa* infection. (A) The representative images of LC3B puncta within MLE‐12 cells (scale bar represents 5 µm, detailed images scale bar represents 2 µm). (B) The expression levels of LC3, PHB2, Parkin, and PINK1 in MLE‐12 cells determined by western blotting and semiquantitative analysis. Data represent means ± SD of three independent experiments, *p < 0.05, **p < 0.01 by two‐way ANOVA with Bonferroni's post hoc test. (C) The expression of PINK1 (left) and PHB2 (right) was analyzed by immunofluorescence in MLE‐12 cells (scale bar represents 5 µm). (D) Mitochondrial membrane potential of MLE‐12 cells measured by JC‐1 fluorescence assay. Data represent means ± SD of six independent experiments, *p < 0.05, **p < 0.01, ***p < 0.001 by two‐way ANOVA with Bonferroni's post hoc test. (E) The representative images of H2DCF‐DA (left) and MitoSOX Red (right) staining in MLE‐12 cells (scale bar represents 5 µm). (F) Relative cytosolic mtDNA levels in MLE‐12 cells. Data represent means ± SD of four independent experiments, *p < 0.05, **p < 0.01, ***p < 0.001, ****p < 0.0001 by two‐way ANOVA with Bonferroni's post hoc test. Ctrl, control; PA, *P. aeruginosa*; siAtg5, *Atg5* siRNA; siNC, control siRNA.

Mitochondria represent a major source of reactive oxygen species (ROS), which can cause cellular damage if not effectively managed [11]. To counteract this, mitophagy—a specialized form of autophagy—plays a crucial role in mitigating mitochondrial ROS (mtROS) by facilitating the removal of damaged mitochondria [12, 13]. To investigate the role of mitophagy in modulating inflammatory responses during *P. aeruginosa* infection, we employed two complementary approaches: pretreatment with the mitochondrial division/mitophagy inhibitor Mdivi‐1 and knockdown of Atg5 in mouse lung tissues via siRNA transfection. Both interventions significantly reduced lung injury and inflammatory cell infiltration compared to controls following *P. aeruginosa* infection (Figure S6), highlighting the critical role of mitophagy in maintaining tissue homeostasis under infection‐induced stress. Moreover, in *Atg5*‐silenced MLE‐12 cells, LC3B puncta, a marker associated with autophagosome formation, displayed decreased accumulation around mitochondria in response to *P. aeruginosa*, indicating a compromised mitophagic process compared to control cells (Figure 5A). Surprisingly, the knockdown of *Atg5* in epithelial cells led to a decrease in the expression of PINK1, Parkin, and PHB2 upon *P. aeruginosa* treatment, implying that the loss of *Atg5* not only disrupts mitophagy by impeding autophagosomal formation but also inhibits both ubiquitin‐dependent and receptor‐mediated mitophagy pathways during infection (Figure 5B,C). Consistent with this, silencing *Atg5* in MLE‐12 cells resulted in aggregated mitochondrial damage, as evidenced by a substantial decrease in mitochondrial membrane potential (Figure 5D) and a significant increase in the production of both total ROS and mtROS during PAO1 infection (Figure 5E). Additionally, considering that mtDNA is released under various pathological conditions, such as oxidative stress and mitochondrial dysfunction [14], we measured cytosolic mtDNA, which was significantly elevated in *Atg5*‐knockdown cells compared to control cells postinfection (Figure 5F). These findings collectively underscore the essential role of ATG5 in mitophagy and the maintenance of mitochondrial homeostasis in alveolar epithelial cells during *P. aeruginosa* infection.

### 2.7 **Loss of** ***Atg5*** **in Epithelial Cells Facilitated Extracellular mtDNA Release During** ***P. aeruginosa*** **Infection, Subsequently Triggering cGAS–STING–NLRP3 Axis Activation in Macrophages**

**FIGURE 6:**
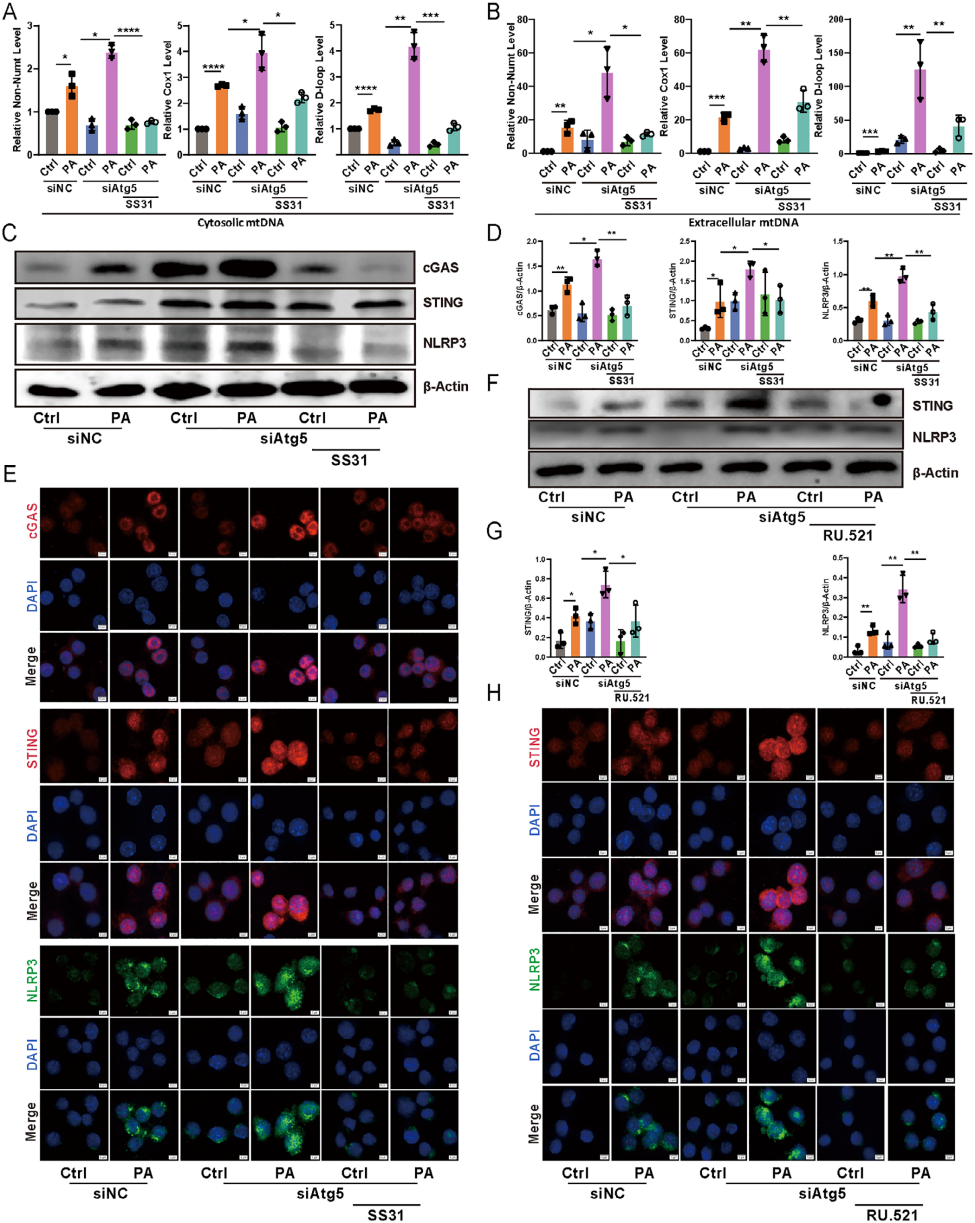
Loss of *Atg5* in epithelial cells facilitated extracellular mtDNA release during *P. aeruginosa* infection, subsequently triggering cGAS–STING–NLRP3 axis activation in macrophages. (A) Relative cytosolic mtDNA levels in MLE‐12 cells. Data represent means ± SD of three independent experiments, *p < 0.05, **p < 0.01, ***p < 0.001, ****p < 0.0001 by two‐way ANOVA with Turkey's post hoc test. (B) Relative extracellular mtDNA levels of MLE‐12 cells. Data represent means ± SD of three independent experiments, *p < 0.05, **p < 0.01, ***p < 0.001 by two‐way ANOVA with Turkey's post hoc test. (C) The expression levels of cGAS, STING, and NLRP3 in RAW264.7 macrophages determined by western blotting. (D) Semiquantitative analysis of (C). Data represent means ± SD of three independent experiments, *p < 0.05, **p < 0.01 by two‐way ANOVA with Turkey's post hoc test. (E) The expression of cGAS (upper), STING (middle), and NLRP3 (lower) was analyzed by immunofluorescence in RAW264.7 macrophages (scale bar represents 5 µm). (F) The expression levels of STING and NLRP3 in RAW264.7 macrophages determined by western blotting. (G) Semiquantitative analysis of (F). Data represent means ± SD of three independent experiments, *p < 0.05, **p < 0.01 by two‐way ANOVA with Turkey's post hoc test. (H) The expression of STING (upper) and NLRP3 (lower) was analyzed by immunofluorescence in RAW264.7 macrophages (scale bar represents 5 µm). Ctrl, control; PA, *P. aeruginosa*; siAtg5, *Atg5* siRNA; siNC, control siRNA.

Recent work highlighted the role of GSDMD in promoting the release of mtDNA from the cytosol into the extracellular space [15]. We speculated that silencing *Atg5* would lead to the accumulation of mtDNA in the extracellular space. Quantitative PCR analysis revealed a significant increase in mtDNA levels in the culture medium supernatant of MLE‐12 cells with *Atg5* knockdown following *P. aeruginosa* infection, compared to control cells (Figure 6B). The escape of mtDNA into the cytosol triggers the activation of the cGAS–STING–NLRP3 axis [16]. The extracellular release of mtDNA may subsequently activate immune cells, offering a plausible explanation for the observed discrepancy where alveolar epithelial *Atg5* deficiency intensified NLRP3 inflammasome activation in lungs, but loss of *Atg5* in alveolar epithelial cells did not. To further investigate this phenomenon, we exposed RAW264.7 murine macrophages to the 0.22 um filtered medium supernatant of *P. aeruginosa*‐infected MLE‐12 cells. Western blotting and immunofluorescence results demonstrated a significant increase in the expression of cGAS, STING, and NLRP3 in macrophages treated with supernatant from *Atg5*‐silenced MLE‐12 cells compared to control cells (Figure 6C–E). Consistent with these findings, our airway epithelial cell delivery experiments showed that lungs receiving Atg5‐knockdown epithelial cells exhibited increased expression of cGAS, STING, and NLRP3 in macrophages following *P. aeruginosa* infection, compared to lungs treated with control siRNA‐transfected epithelial cells (Figure S7). To validate these findings, we employed the mitochondria‐targeted peptide SS‐31, known for neutralizing ROS and inhibiting mtDNA release [17]. Indeed, SS‐31 markedly decreased mtDNA levels in both the cytoplasm and culture medium supernatant of *Atg5*‐knockdown epithelial cells during *P. aeruginosa* infection (Figure 6A,B). Furthermore, the heightened expression of cGAS, STING, and NLRP3 in macrophages treated with supernatant from *P. aeruginosa*‐infected Atg5‐silenced MLE‐12 cells was significantly mitigated by SS‐31 treatment of the epithelial cells (Figure 6C–E). In addition, we used RU.521, a selective inhibitor targeting the catalytic site of cGAS [18]. This inhibitor significantly suppressed downstream STING and NLRP3 expression in macrophages treated with supernatant from *Atg5*‐silenced MLE‐12 cells infected with *P. aeruginosa* (Figure 6F–H). This observation suggests that inhibiting cGAS activity, which impairs cGAMP production, indirectly affects STING expression. This suggests a possible feedback mechanism wherein the downstream signaling components of the cGAS‐STING pathway are modulated by upstream inhibition. Altogether, these results underscore the pivotal role of ATG5 in controlling extracellular mtDNA accumulation from alveolar epithelial cells and subsequent cGAS–STING–NLRP3 signaling activation in macrophages during *P. aeruginosa* infection.

## 3 Discussion

The investigation into the essential role of autophagy in *P. aeruginosa* infection has yielded insights into the intricate landscape of host–pathogen interactions and immune defense mechanisms [1, 19]. Our bulk RNA‐seq results are consistent with previous studies that autophagy is an important characteristic of lungs upon *P. aeruginosa* invasion [20, 21]. Of note, employing single‐cell transcriptomics, our investigation may advance current understanding by revealing that AEC2 manifests the strongest activity in autophagy formation during *P. aeruginosa* infection, spotlighting a cell‐type‐specific engagement of autophagy. The cell‐type specificity observed underscores the intricate nature of autophagy engagement in the pulmonary infection, accentuating the imperative to dissect these responses within the epithelial cell milieu. Our study involving conditional *Atg5* knockout mice emphasizes the crucial role of epithelial autophagy in the face of *P. aeruginosa* infection. The observed reduction in survival and increased bacterial dissemination echoes earlier study where autophagy induced by type III secretion system (T3SS) toxins was recognized for enhancing the clearance of this pathogen from human corneal epithelial cells [22]. Similarly, autophagy has been identified as a protective mechanism, safeguarding AEC2 against *Mycobacterium tuberculosis* infection [23]. In the context of fungal infection, ATG5 and ATG16L1 contribute to plasma membrane repair through lysosomal exocytosis, safeguarding epithelial cells against *Candida albicans* (*C. albicans)* [24]. Vaginal epithelial cells with active autophagy exhibited resilience against *C. albicans* infection, while those with defective autophagy succumbed to the onslaught [25]. Our study extends these observations, revealing worsened lung injury, hyperactivated inflammasomes, heightened pyroptosis and inflammatory responses, and aggravated epithelial barrier dysregulation in epithelial *Atg5*‐deficient mice following *P. aeruginosa* infection, aligning with our earlier reports on the severity of lung injury and enhanced inflammasome activation in *Atg7* ^−/−^ mice [7]. This collective evidence supports the pivotal role of epithelial ATG5‐mediated autophagy in shielding against microbial‐induced pulmonary injury through mechanisms, such as bacterial clearance, modulation of proinflammatory signaling, and maintenance of barrier integrity. However, this protective role contrasts with the reported response to viral infections. Contrary to bacterial and fungal infections, respiratory syncytial virus replication is facilitated by autophagy in epithelial cells [26]. Another study points toward autophagy promoting H9N2 virus replication in alveolar epithelial cells by modulating oxidative stress via the Akt/TSC2/mTOR signaling pathway [27]. These distinctions underscore the pathogen‐dependent role of epithelial autophagy in infections, urging further exploration to unravel the intricacies of these divergent responses and inform potential therapeutic interventions.

In a recent study, *P. aeruginosa* biofilm was implicated in the activation of NLRP3 inflammasomes in macrophages [28]. Notably, NLRP3 was found to selectively drive IL‐1β secretion in *P. aeruginosa*‐infected neutrophils, thereby regulating the severity of infection [29]. The T3SS and the Type I CRISPR‐Cas system were found to play a pivotal role in promoting the assembly and activation of the NLRC4 inflammasome [1]. This process occurs in macrophages that are subjected to the challenge of *P. aeruginosa* and is finely orchestrated by the autophagy. Our prior investigations revealed that the silencing of *Atg7* in alveolar macrophages amplifies NLRC4 inflammasome activation and pyroptosis subsequent to *P. aeruginosa* infection [30]. However, it is worth noting that these insights primarily stem from experiments involving immune cells. Herein, our study unveils an unexpected facet of ATG5 involvement, highlighting its role in activating the noncanonical caspase‐11 inflammasome in lung epithelial cells during *P. aeruginosa* stimulation, while showing no influence on NLRP3 and NLRC4 activation. This discovery adds complexity to our comprehension of autophagy regulation in inflammasome activation during bacterial infections. Caspase‐11, directly recognizing bacterial lipopolysaccharide, cleaves GSDMD, initiating both pyroptosis and NLRP3‐dependent caspase‐1 activation and IL‐1β maturation in bone‐marrow‐derived macrophages [31]. Intriguingly, our results demonstrate that while GSDMD cleavage increased alongside caspase‐11 activation in alveolar epithelial cells with *Atg5* loss upon *P. aeruginosa* infection, no concurrent activation of caspase‐1 or maturation of IL‐1β was observed. These findings align with recent work by Wang et al., who elucidated the roles of caspase‐1 and caspase‐11 in *Burkholderia pseudomallei* infection [32]. They found that caspase‐1 predominantly governs the production of IL‐1β and IL‐18, as well as pyroptosis in infected macrophages, whereas caspase‐11 orchestrates pyroptotic cell death specifically in infected lung epithelial cells. Similarly, the observed differences between siRNA‐mediated *Atg5* knockdown in epithelial cells and genetic *Atg5* deficiency in *Atg5* ^ΔAEC2^ mice likely stem from inherent differences in these experimental models. The in vivo model incorporates systemic and microenvironmental factors, including interactions with immune cells and other stromal cells, which may amplify the effects of ATG5 deficiency and contribute to the observed discrepancies. Nevertheless, further studies are imperative to elucidate the regulatory role and underlying mechanisms of ATG5‐mediated autophagy in governing noncanonical caspase‐11 inflammasome‐mediated GSDMD activation in alveolar epithelial cells under bacterial challenges.

Upon cleavage, the N‐terminal domain of gasdermins infiltrates cellular membranes, forming oligomeric membrane pores that lead to membrane rupture and ensuing pyroptotic cell death. These membrane pores enable the discharge of activated cytokines or other damage‐associated molecular patterns, intensifying the subsequent inflammatory responses [33]. H7N9 virus‐induced pyroptosis in alveolar epithelial cells involves gasdermin E, resulting in the release of cytosolic contents that precipitate a cytokine storm [34]. In our study, the loss of *Atg5* in epithelial cells facilitated the extracellular release of mtDNA during *P. aeruginosa* challenge. Our observation aligns with recent studies demonstrating that the N‐terminal domain of GSDMD, in permeabilizing the plasma membrane, plays a crucial role in releasing mtDNA. This process occurs in two steps: an initial relocation to the cytosol and a subsequent release into the cellular milieu following severe plasma membrane destabilization during pyroptosis [15]. Furthermore, our observation that the extracellular release of mtDNA from epithelial cells subsequently activates the cGAS–STING–NLRP3 axis in macrophages during *P. aeruginosa* infection. Similar mechanistic insights have been uncovered in the context of XBP1 deficiency, where impaired mitophagy facilitates the extracellular release of mtDNA. This occurs through hepatocyte pyroptosis, subsequently activating the macrophage STING pathway during acute liver injury [35]. This provides a possible explanation for our observed paradox: while alveolar epithelial *Atg5* deficiency heightened NLRP3 inflammasome activation in the infected lungs, the loss of *Atg5* did not elicit the same response in alveolar epithelial cells. Collectively, these findings reveal a pathway of mtDNA release, offering insight into the intercellular regulatory processes dependent on epithelial ATG5 and their implications for immune responses during microbial challenges. However, another consideration is the potential involvement of additional danger‐associated molecular patterns (DAMPs) in the activation of the cGAS/STING/NLRP3 signaling pathway observed in this study. It is important to acknowledge that other DAMPs released by dying epithelial cells could also contribute to this activation. Future studies are warranted to explore the relative contributions of other DAMPs alongside mtDNA in this context, offering a quite comprehensive understanding of the molecular signals driving the cGAS/STING/NLRP3 pathway during *P. aeruginosa* infection.

Mitochondrial damage serves as a critical trigger for *P. aeruginosa*‐induced activation of inflammasomes, a process meticulously regulated by autophagy [36]. The failure of autophagosome formation, a key player in cellular quality control, results in the aggregation of mitochondrial damage [37]. Our investigations into the impact of *Atg5* deficiency in epithelial cells upon stimulation by *P. aeruginosa* have unveiled an impairment in mitophagy, culminating in the aggregation of mitochondrial damage. This compromised condition is marked by a diminished mitochondrial membrane potential, heightened mtROS production, and the accumulation of cytoplasmic mtDNA. These findings resonate with prior research underlining the pivotal role of ATG5 in preserving mitochondrial homeostasis during microbial challenges [38]. However, an unforeseen revelation unfolded as a reduction in the expression of pivotal mitophagy regulators, specifically PINK1, Parkin, and PHB2, was noted in *Atg5*‐silenced epithelial cells exposed to *P. aeruginosa*. This unexpected decrease implies that the loss of *Atg5* not only hampers autophagosomal formation but disrupts both ubiquitin‐dependent and receptor‐mediated mitophagy pathways. While JC‐1 staining provided valuable insights into mitochondrial membrane potential, it offers a limited perspective on overall mitochondrial function. Future endeavors aimed at unraveling the molecular interactions involving ATG5, PINK1, Parkin, and PHB2, combined with more comprehensive functional assays, hold the potential to provide insights into the intricate role of ATG5 in mitophagy, particularly in the context of bacterial infections.

**FIGURE 7:**
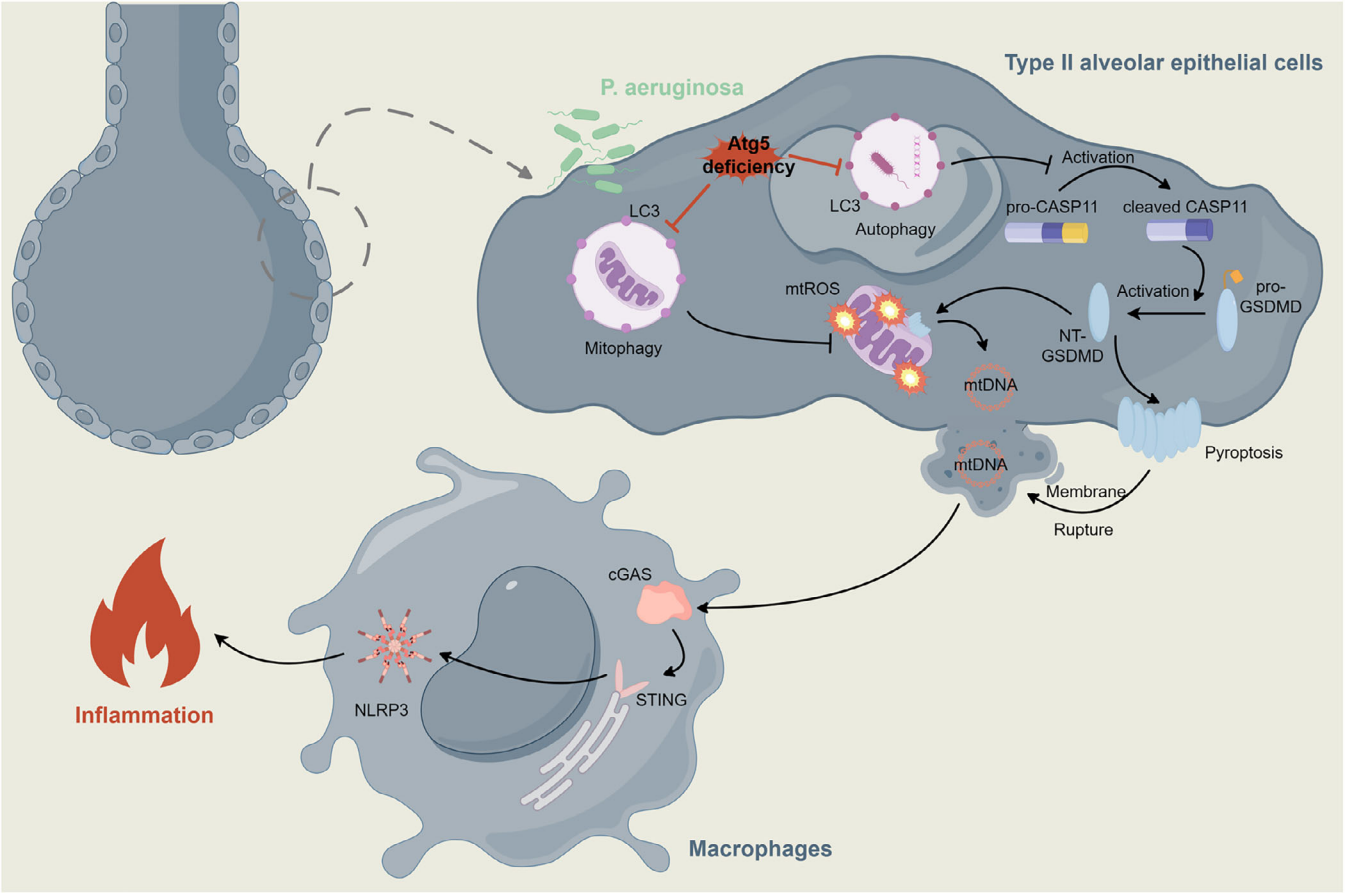
Epithelial *Atg5* deficiency intensifies caspase‐11 activation, fueling extracellular mtDNA release to activate cGAS–STING–NLRP3 axis in macrophages during *Pseudomonas* infection.

In conclusion, our work illuminates the essential role of epithelial ATG5 in the defense against *P. aeruginosa*. *Atg5* deficiency heightens vulnerability, compromising the clearance of bacteria and exacerbating systemic dissemination. The aggravated tissue inflammatory response, involving the activation of the AKT/PI3K/NF‐κB axis, inflammasomes, and pyroptosis, culminates in severe lung injury. Mechanistically, the interference with mitophagy due to *Atg5* loss in epithelial cells intensifies mitochondrial damage. This, combined with the enhanced activation of GSDMD mediated by the noncanonical caspase‐11 inflammasome, amplifies the extracellular release of mtDNA, triggering cGAS—STING–NLRP3 signaling in macrophages (Figure 7). Our findings broaden the understanding of microbial infections, particularly those involving bacteria that have evolved mechanisms to hijack autophagosome formation, evading host elimination. The compromised autophagy observed in *Atg5*‐deficient conditions may contribute to a protracted inflammatory state and increased tissue injury. Targeting autophagy pathways might potentially disrupt bacterial survival mechanisms and alleviate the excessive inflammation and injury associated with certain infections, paving the way for innovative therapeutic interventions.

In acknowledging the limitations of our study, it is essential to note that we primarily focused on the role of ATG5‐dependent autophagy in the host defense against *P. aeruginosa*. Future investigations should investigate the complexities of ATG5‐independent autophagy to provide a more comprehensive understanding of the host's multifaceted responses to microbial challenges. Moreover, this study did not address whether *P. aeruginosa* naturally evades host defenses by targeting or blocking the autophagic pathway in epithelial cells. In addition, the findings of this study are primarily based on murine models and in vitro systems, which, while highly informative, may not fully capture the complexity of human pulmonary infections. Future research incorporating human tissue models or clinical samples will be essential to validate and extend the translational relevance of our findings.

## 4 Materials and Methods

### 4.1 Mice

Six to eight weeks old C57BL/6 mice were purchased from the Jackson Laboratory. The *Atg5* ^ΔAEC2^ mice were generated on a C57BL/6J background by crossing *Atg5* ^f/f^ mice with *Sftpc* ^CreER^ mice to produce *Sftpc* ^CreER^;*Atg5* ^f/f^ mice [39]. To induce Atg5 knockout specifically in AT2 cells, tamoxifen was administered intraperitoneally at a dose of 50 mg/kg once daily for 5 consecutive days. The mice were bred and housed at the University of North Dakota, with all animal procedures approved by the University of North Dakota Institutional Animal Care and Use Committee (2210‐0045) and conducted in compliance with animal care and institutional protocols.

### 4.2 Cell Culture, Transfection, and Treatment

Murine epithelial cells (MLE‐12) and macrophage cells (RAW264.7) were acquired from the American Type Culture Collection. RAW264.7 cells were cultured in high glucose Dulbecco's modified Eagle medium supplemented with 10% fetal bovine serum and 100 U/mL penicillin–streptomycin antibiotics, maintained in a 37°C incubator with 5% CO_2_. MLE‐12 cells were cultured in Dulbecco's modified Eagle medium/nutrient mixture F‐12 medium. Transfection of MLE‐12 cells with small interfering RNAs was carried out using RNAiMAX (Invitrogen), following the manufacturer's instructions. The *Atg5* siRNA was procured from Santa Cruz Biotechnology.

### 4.3 Bacteria Preparation and Infection Experiments

*P. aeruginosa* strain PAO1 was graciously provided by Dr. S. Lory (Harvard Medical School, Boston, MA, USA). The bacteria were cultivated in lysogeny broth (LB) medium at a constant temperature of 37°C with shaking at 220 rpm until reaching an OD_600_ of 0.6–0.8. For in vivo experiments, mice were anesthetized with ketamine (80 mg/kg) and xylazine (10 mg/kg), then intranasally instilled with 1 × 10^7^ clonal‐forming units (CFUs) of PAO1 in 30 µL phosphate‐buffered saline (PBS). The health status of mice was closely monitored, and euthanasia was performed upon observing moribund conditions to generate survival curves using Kaplan–Meier methods and to facilitate additional assessments.

For airway epithelial cell delivery experiments, we utilized a previously established intratracheal delivery model in mice, which achieves a cell‐retention efficiency exceeding 10% [40]. C57BL/6J mice were administered 20 µL of 2% polidocanol (PDOC; Sigma‐Aldrich, St. Louis, MO, USA) in PBS intratracheally to prepare the lungs. After 24 h, MLE‐12 cells transfected with Atg5 siRNA or siNC (1 × 10^7^ cells per mouse) were delivered via intratracheal injection. The next day, mice were infected with *P. aeruginosa* (PAO1) and lung tissues were harvested 24 h postinfection for analysis. For inhibitor experiments, mice were pretreated with the AKT inhibitor AKTi ½ (20 mg/kg) or the NF‐κB inhibitor JSH‐23 (40 mg/kg) via intraperitoneal injection for 2 consecutive days prior to PAO1 infection. Lung tissues were collected 24 h after infection for further analysis.

For Atg5 knockdown in vivo experiments, 20 µg of Atg5 siRNA or siNC was mixed with 7.5 µL of Entranster‐in vivo transfection reagent (Engreen Biosystem) and 7.5 µL of saline to a total volume of 30 µL. The mixture was administered intranasally to C57BL/6J mice. After 24 h, mice were infected with PAO1 (1 × 10⁷ CFU). Lung tissues were harvested 24 h postinfection for analysis. For mitophagy inhibition experiments, mice were pretreated with the mitochondrial division/mitophagy inhibitor Mdivi‐1 (50 mg/kg) via gavage for 5 consecutive days. Following pretreatment, mice were infected with PAO1 (1 × 10⁷ CFU). Lung tissues were collected 24 h postinfection for further analysis.

In the epithelial infection experiments, MLE‐12 cells were infected with PAO1 at an MOI of 20 for 2 h after cell fusion reached 60%–80%. To assess the impact of SS‐31, MLE‐12 cells were treated with 200 nM SS‐31 along with PAO1. Following the infection, supernatant was collected, subjected to centrifugation, and bacteria were removed using a 0.22 µm filter. The resulting supernatant was then utilized to culture RAW264.7 cells for 24 h. For RU.521, RAW264.7 cells were pretreated with 20 uM RU.521 for 2 h, followed by culturing with the supernatant of PAO1‐treated MLE‐12 cell medium for 24 h.

### 4.4 Bacterial Burden Assay

Lung, spleen, and liver homogenates, BALF, and blood collected from mice were appropriately diluted in PBS to various concentrations. Subsequently, the diluted samples were evenly spread on LB agar dishes. These dishes were incubated overnight in a 37°C environment, after which bacterial colonies were counted for analysis.

### 4.5 Histological Analysis

Lung tissues were fixed in 4% paraformaldehyde (PFA), followed by a 48‐h dehydration in 75% alcohol at 4°C. Subsequently, the tissues were embedded in paraffin using a routine histological procedure. H&E staining, following the standard staining protocol, was then performed for histological examination. The lung injury was scored in a blinded manner according to previous method [41].

### 4.6 Mitochondrial Membrane Potential Assay

The assessment of mitochondrial membrane potential was conducted employing the JC‐1 Mitochondrial Membrane Potential Assay Kit (Cayman Chemical), following the manufacturer's guidelines. In brief, MLE‐12 cells, infected with PAO1 for 2 h as described earlier, were treated with 1 µg/mL of JC‐1 and incubated for 30 min. Fluorescence intensity was quantified using a fluorescence plate reader at 560‐nm excitation with a 595‐nm emission filter and at 485‐nm excitation with a 535‐nm emission filter.

### 4.7 Measurement of Reactive Oxygen Species

Intracellular ROS were assessed using H_2_DCF‐DA staining solution (Invitrogen), following the manufacturer's stipulated guidelines. mtROS levels were quantified employing MitoSOX Red Mitochondrial Superoxide Indicators (Invitrogen). MLE‐12 cells, subjected to the specified treatments, were incubated with 0.5 µM MitoSOX reagent for 30 min. Following two washes, fluorescence emissions were recorded using confocal laser scanning microscopy at 595 nm.

### 4.8 Immunoblotting

Lung tissues or whole‐cell lysates were prepared in RIPA buffer (Thermo Fisher), enriched with a protease inhibitor cocktail and a phosphatase inhibitor cocktail (Invitrogen). Protein concentrations were quantified using the BCA Protein Assay Kit (Thermo Fisher). Subsequently, 20 µg of protein were electrophoretically separated on SDS–PAGE gels and transferred onto PVDF membranes. The membranes were then blocked in 5% skim milk in 1 × TBST for 1 h, followed by an incubation with primary antibodies (1:1000 dilution; ATG5, TNF‐α, IL‐1β, PI3K, p‐PI3K, AKT, p‐AKT, p‐P65, CASP1, CASP11, NLRP3, GSDMD, PINK1, Parkin, and PHB2 from Cell Signaling Technology; NLRC4 from ABclonal; P65 from Invitrogen; STAT3 and p‐STAT3 from Santa Cruz Biotechnology; ZO‐1 from Abcam; cGAS and STING from Proteintech) at 4°C overnight. Subsequent incubation involved secondary antibodies conjugated to HRP (ABclonal, 1:10,000 dilution). Signal detection was achieved using an enhanced chemiluminescence detection kit (Cytiva).

### 4.9 Immunofluorescence and Confocal Microscopy

Lung sections (5 µm thickness) and cells cultured on coverslips underwent fixation in 4% PFA, permeabilization in 0.1% Triton X‐100, and blocking in PBS supplemented with 10% goat serum. Primary antibodies (1:200 dilution; ZO‐1 from Abcam; LC3B, NLRP3, PINK1, PHB2, cleaved GSDMD, and cleaved CASP1 from Cell Signaling Technology; cGAS and STING from Proteintech) were incubated in the blocking buffer at 4°C overnight. Subsequently, Goat anti‐Mouse Alexa Fluor 488 Antibody, Goat anti‐Mouse Alexa Fluor 594 Antibody, Goat anti‐Rabbit Alexa Fluor 488 Antibody, and Goat anti‐Rabbit Alexa Fluor 555 Antibody (Invitrogen; 1:1000 dilution) were employed to bind primary antibodies at room temperature for 1 h. Nuclei were counterstained with DAPI, and images were acquired using an Olympus FV3000 confocal microscope. For mitochondrial labeling, cells were incubated with MitoTracker Red (Invitrogen, M7512).

### 4.10 Enzyme‐Linked Immunosorbent Assay

Paired antibodies (capture and detection) and standard recombinant mouse IL‐1β and TNF‐α were used to determine mouse cytokine concentrations in BALF according to manufacturer's instructions.

### 4.11 Measurement of mtDNA

MLE‐12 cells underwent PBS washing and harvesting, with 10% reserved for whole‐cell DNA extraction. The remaining cells were resuspended in prechilled mitochondrial extraction buffer. Subsequently, these cells were subjected to 20 passes through a 25‐G syringe on ice and two‐step centrifugation to facilitate separation of mitochondria and cytosolic fractions [42]. The purification of DNA from whole‐cell extracts, cytosolic fractions, or culture media was carried out using the DNA Extraction Kit (TIANGEN) per manufacturer's instructions. mtDNA was quantified by qPCR using primers specific for the mitochondrial D‐loop region (Forward: 5′‐AATCTACCATCCTCCGTGAAACC‐3′, Reverse: 5′‐TCAGTTTAGCTACCCCCAAGTTTAA‐3′), cytochrome *c* oxidase (Forward: 5′‐GCCCCAGATATAGCATTCCC‐3′, Reverse: 5′‐GTTCATCCTGTTCCTGCTCC‐3′) or a specific region of mtDNA that is not inserted into nuclear DNA (Forward: 5′‐CTAGAAACCCCGAAACCAAA‐3′, Reverse: 5′‐CCAGCTATCACCAAGCTCGT‐3′). Nuclear DNA encoding telomerase reverse transcriptase (Forward: 5′‐CTAGCTCATGTGTCAAGACCCTCTT‐3′, Reverse: 5′‐CTAGCTCATGTGTCAAGACCCTCTT‐3′), 18S ribosomal RNA (Forward: 5′‐TAGAGGGACAAGTGGCGTTC‐3′, Reverse: 5′‐CGCTGAGCCAGTCAGTGT‐3′), and β2 microglobulin (Forward: 5′‐ATGGGAAGCCGAACATACTG‐3′, Reverse: 5′‐CAGTCTCAGTGGGGGTGAAT‐3′) was used for normalization.

### 4.12 Transcriptome Analysis

The transcriptome library was prepared using the TruSeq RNA Sample Preparation Kit (Illumina, San Diego, CA, USA) with 5 µg of total RNA as input. PolyA selection was performed using oligo(dT) beads to isolate mRNA, which was subsequently fragmented. Complementary DNA (cDNA) synthesis was conducted using the SuperScript Double‐Stranded cDNA Synthesis Kit with random hexamer primers (Invitrogen, CA). The resulting cDNA underwent end‐repair, phosphorylation, and addition of an “A” base in accordance with Illumina's library preparation protocol. Library size selection was performed on a 2% low range ultra agarose gel, followed by amplification using Phusion DNA Polymerase with 15 PCR cycles. Paired‐end sequencing was carried out on the Illumina HiSeq X Ten platform by Majorbio (Shanghai, China). Raw paired‐end reads were trimmed and quality‐checked using SeqPrep and Sickle with default parameters. Clean reads were then aligned to the reference genome in orientation mode using TopHat (version 2.0.0). ssGSEA was performed via GSVA package (1.42.0; method = “ssgsea,” kcdf = “Poisson”) [43]. PPI analysis was performed utilizing the STRING database. Autophagy‐related signatures are available in Table S1.

SnRNA‐seq was performed by SingulOmics Corporation (https://singulomics.com/). After quality control (nFeature_RNA > 400 and nFeature_RNA < 4000 and percent.mt < 5) and normalization with SCTransform(), integration was performed by FindIntegrationAnchors() and IntegrateData() function in Seurat (v4.1.1) [44, 45]. Principal components analysis and clustering were conducted with 30 PCs and a resolution of 0.4 in FindClusters(). Single‐cell differential expression gene (DEGs) analyses were performed via MAST R package (1.20.0) [46]. Likelihood ratio tests between the full and reduced model formulas were used to identify DEGs (Benjamini and Hochberg method was performed for multiple testing corrections, FDR < 0.05). Autophagy formation gene signature is available in Table S1. Calculation and visualization of AUC scores were conducted via AUCell package (1.16.0) in R (4.1.0) [47, 48].

### 4.13 Statistical Analysis

All data were presented as mean ± SD from at least three independent experiments. Statistical analyses were done using Graphpad Prism (8.0.2). Multiple group comparisons were analyzed using one‐way ANOVA or two‐way ANOVA. Two group comparisons were analyzed by nonpaired *t*‐test. Statistical significance was defined as a *p* value < 0.05.

## Author Contributions

Junyi Wang and Min Wu designed research; Junyi Wang, Lei Zhang, Yingying Liu, Yao Liu, Anying Xiong, and Qin Ran performed experiments; Junyi Wang, Guoping Li, Colin Combs, and Min Wu wrote the manuscript; Xiang He, Vincent Kam Wai Wong, Colin Combs, and Guoping Li provided regents and critical suggestions. All the authors contributed to the article and approved the submitted version.

## Ethics Statement

All animal procedures approved by the University of North Dakota Institutional Animal Care and Use Committee (2210‐0045).

## Conflicts of Interest

The authors declare no conflicts of interest.

## Supporting information



Supporting Information

## Data Availability

All data supporting the findings of this study are available from the authors upon reasonable request. The RNA‐seq and SnRNA‐seq data were deposited into the National Genomics Data Center of China National Center for Bioinformation under accession number CRA024835.

## References

[1] S. Qin , W. Xiao , C. Zhou , et al., “ *Pseudomonas aeruginosa*: Pathogenesis, Virulence Factors, Antibiotic Resistance, Interaction With Host, Technology Advances and Emerging Therapeutics,” Signal Transduction and Targeted Therapy 7, no. 1 (2022): 199. 35752612 10.1038/s41392-022-01056-1 PMC9233671

[2] D. M. P. De Oliveira , B. M. Forde , T. J. Kidd , et al., “Antimicrobial Resistance in ESKAPE Pathogens,” Clinical Microbiology Reviews 33, no. 3 (2020): e00181‐19. 10.1128/CMR.00181-19 PMC7227449 32404435

[3] E. Tacconelli , E. Carrara , A. Savoldi , et al., “Discovery, Research, and Development of New Antibiotics: The WHO Priority List of Antibiotic‐Resistant Bacteria and Tuberculosis,” Lancet Infectious Diseases 18, no. 3 (2018): 318–327. 29276051 10.1016/S1473-3099(17)30753-3

[4] N. Mizushima and B. Levine , “Autophagy in Human Diseases,” New England Journal of Medicine 383, no. 16 (2020): 1564–1576. 33053285 10.1056/NEJMra2022774

[5] J. Cahoon , D. Yang , and P. Wang , “The Noncanonical Inflammasome in Health and Disease,” Infectious Medicine (Beijing) 1, no. 3 (2022): 208–216. 10.1016/j.imj.2022.09.001 PMC10699704 38077630

[6] B. Aylan , E. M. Bernard , E. Pellegrino , et al., “ATG7 and ATG14 Restrict Cytosolic and Phagosomal *Mycobacterium tuberculosis* Replication in Human Macrophages,” Nature Microbiology 8, no. 5 (2023): 803–818. 10.1038/s41564-023-01335-9 PMC10159855 36959508

[7] Q. Pu , C. Gan , R. Li , et al., “Atg7 Deficiency Intensifies Inflammasome Activation and Pyroptosis in *Pseudomonas* Sepsis,” Journal of Immunology 198, no. 8 (2017): 3205–3213. 10.4049/jimmunol.1601196 PMC5382979 28258192

[8] V. L. Campodonico , M. Gadjeva , C. Paradis‐Bleau , A. Uluer , and G. B. Pier , “Airway Epithelial Control of *Pseudomonas aeruginosa* Infection in Cystic Fibrosis,” Trends in Molecular Medicine 14, no. 3 (2008): 120–133. 18262467 10.1016/j.molmed.2008.01.002 PMC3697050

[9] J. Z. Cui , Z. H. Chew , and L. H. K. Lim , “New Insights Into Nucleic Acid Sensor AIM2: The Potential Benefit in Targeted Therapy for Cancer,” Pharmacological Research 200 (2024): 107079. 38272334 10.1016/j.phrs.2024.107079

[10] X. Jin , Y. Ma , D. Liu , and Y. Huang , “Role of Pyroptosis in the Pathogenesis and Treatment of Diseases,” MedComm 4, no. 3 (2023): e249. 37125240 10.1002/mco2.249 PMC10130418

[11] G. V. Raghuram , B. K. Tripathy , K. Avadhani , et al., “Cell‐Free Chromatin Particles Released From Dying Cells Inflict Mitochondrial Damage and ROS Production in Living Cells,” Cell Death Discovery 10, no. 1 (2024): 30. 38225229 10.1038/s41420-023-01728-z PMC10789803

[12] S. Wang , H. Long , L. Hou , et al., “The Mitophagy Pathway and Its Implications in human Diseases,” Signal Transduction and Targeted Therapy 8, no. 1 (2023): 304. 37582956 10.1038/s41392-023-01503-7 PMC10427715

[13] J. Liu , J. Wang , A. Xiong , et al., “Mitochondrial Quality Control in Lung Diseases: Current Research and Future Directions,” Frontiers in Physiology 14 (2023): 1236651. 37538379 10.3389/fphys.2023.1236651 PMC10395103

[14] L. Chen , M. Zhou , H. Li , et al., “Mitochondrial Heterogeneity in Diseases,” Signal Transduction and Targeted Therapy 8, no. 1 (2023): 311. 37607925 10.1038/s41392-023-01546-w PMC10444818

[15] C. de Torre‐Minguela , A. I. Gomez , I. Couillin , and P. Pelegrin , “Gasdermins Mediate Cellular Release of Mitochondrial DNA During Pyroptosis and Apoptosis,” FASEB Journal 35, no. 8 ( 2021): e21757. 34233045 10.1096/fj.202100085R

[16] W. Zhang , G. Li , R. Luo , et al., “Cytosolic Escape of Mitochondrial DNA Triggers cGAS‐STING‐NLRP3 Axis‐Dependent Nucleus Pulposus Cell Pyroptosis,” Experimental & Molecular Medicine 54, no. 2 (2022): 129–142. 35145201 10.1038/s12276-022-00729-9 PMC8894389

[17] Y. Wu , C. Hao , G. Han , et al., “SS‐31 Ameliorates Hepatic Injury in Rats Subjected to Severe Burns plus Delayed Resuscitation via Inhibiting the mtDNA/STING Pathway in Kupffer Cells,” Biochemical and Biophysical Research Communications 546 (2021): 138–144. 33582556 10.1016/j.bbrc.2021.01.110

[18] J. Vincent , C. Adura , P. Gao , et al., “Small Molecule Inhibition of cGAS Reduces Interferon Expression in Primary Macrophages From Autoimmune Mice,” Nature Communications 8, no. 1 (2017): 750. 10.1038/s41467-017-00833-9 PMC5622107 28963528

[19] C. G. Zou , Y. C. Ma , L. L. Dai , and K. Q. Zhang , “Autophagy Protects *C. elegans* Against Necrosis During *Pseudomonas aeruginosa* Infection,” PNAS 111, no. 34 (2014): 12480–12485. 25114220 10.1073/pnas.1405032111 PMC4151725

[20] Q. Deng , Y. Wang , Y. Zhang , et al., “ *Pseudomonas aeruginosa* Triggers Macrophage Autophagy to Escape Intracellular Killing by Activation of the NLRP3 Inflammasome,” Infection and Immunity 84, no. 1 (2016): 56–66. 26467446 10.1128/IAI.00945-15 PMC4694000

[21] P. Zhu , H. Bu , S. Tan , et al., “A Novel Cochlioquinone Derivative, CoB,” Journal of Immunology 1, no. 5 (2020): 1293–1305. 10.4049/jimmunol.1901346 32747503

[22] V. Mohankumar , S. Ramalingam , G. P. Chidambaranathan , and L. Prajna , “Autophagy Induced by Type III Secretion System Toxins Enhances Clearance of *Pseudomonas aeruginosa* From human Corneal Epithelial Cells,” Biochemical and Biophysical Research Communications 503, no. 3 (2018): 1510–1515. 30031608 10.1016/j.bbrc.2018.07.071

[23] X. G. Guo , T. X. Ji , Y. Xia , and Y. Y. Ma , “Autophagy Protects Type II Alveolar Epithelial Cells From *Mycobacterium tuberculosis* Infection,” Biochemical and Biophysical Research Communications 432, no. 2 (2013): 308. 23396060 10.1016/j.bbrc.2013.01.111

[24] P. Lapaquette , A. Ducreux , L. Basmaciyan , et al., “Membrane Protective Role of Autophagic Machinery During Infection of Epithelial Cells by *Candida albicans* ,” Gut Microbes 14, no. 1 (2022): 2004798. 35086419 10.1080/19490976.2021.2004798 PMC8803057

[25] A. Shroff and K. V. R. Reddy , “Autophagy Gene ATG5 Knockdown Upregulates Apoptotic Cell Death During *Candida albicans* Infection in Human Vaginal Epithelial Cells,” American Journal of Reproductive Immunology 80, no. 6 (2018): e13056. 30303264 10.1111/aji.13056

[26] M. Li , J. Li , R. Zeng , et al., “Respiratory Syncytial Virus Replication Is Promoted by Autophagy‐Mediated Inhibition of Apoptosis,” Journal of Virology 92, no. 8 (2018): e02193‐17. 10.1128/JVI.02193-17 PMC5874425 29386287

[27] R. H. Zhang , H. L. Zhang , P. Y. Li , et al., “Autophagy Is Involved in the Replication of H9N2 Influenza Virus via the Regulation of Oxidative Stress in Alveolar Epithelial Cells,” Virology Journal 18, no. 1 (2021): 22. 33461581 10.1186/s12985-020-01484-x PMC7814439

[28] Q. Tan , Q. Ai , Y. He , F. Li , and J. Yu , “ *P. aeruginosa* Biofilm Activates the NLRP3 Inflammasomes In Vitro,” Microbial Pathogenesis 164 (2022): 105379. 35038547 10.1016/j.micpath.2021.105379

[29] M. S. Minns , K. Liboro , T. S. Lima , et al., “NLRP3 Selectively Drives IL‐1Beta Secretion by *Pseudomonas aeruginosa* Infected Neutrophils and Regulates Corneal Disease Severity,” Nature Communications 14, no. 1 (2023): 5832. 10.1038/s41467-023-41391-7 PMC10511713 37730693

[30] X. Li , Y. Ye , X. Zhou , C. Huang , and M. Wu , “Atg7 Enhances Host Defense Against Infection via Downregulation of Superoxide but Upregulation of Nitric Oxide,” Journal of Immunology 194, no. 3 (2015): 1112–1121. 10.4049/jimmunol.1401958 PMC4409144 25535282

[31] N. Kayagaki , I. B. Stowe , B. L. Lee , et al., “Caspase‐11 Cleaves Gasdermin D for Non‐Canonical Inflammasome Signalling,” Nature 526, no. 7575 (2015): 666–671. 26375259 10.1038/nature15541

[32] J. Wang , M. Sahoo , L. Lantier , et al., “Caspase‐11‐Dependent Pyroptosis of Lung Epithelial Cells Protects From Melioidosis While Caspase‐1 Mediates Macrophage Pyroptosis and Production of IL‐18,” PLoS Pathogens 14, no. 5 (2018): e1007105. 29791511 10.1371/journal.ppat.1007105 PMC5988316

[33] J. Shi , Y. Zhao , K. Wang , et al., “Cleavage of GSDMD by Inflammatory Caspases Determines Pyroptotic Cell Death,” Nature 526, no. 7575 (2015): 660–665. 26375003 10.1038/nature15514

[34] X. Wan , J. Li , Y. Wang , et al., “H7N9 Virus Infection Triggers Lethal Cytokine Storm by Activating Gasdermin E‐Mediated Pyroptosis of Lung Alveolar Epithelial Cells,” National Science Review 9, no. 1 (2022): nwab137. 35087672 10.1093/nsr/nwab137 PMC8788236

[35] Z. Liu , M. Wang , X. Wang , et al., “XBP1 Deficiency Promotes Hepatocyte Pyroptosis by Impairing Mitophagy to Activate mtDNA‐cGAS‐STING Signaling in Macrophages During Acute Liver Injury,” Redox Biology 52 (2022): 102305. 35367811 10.1016/j.redox.2022.102305 PMC8971356

[36] M. S. Jabir , L. Hopkins , N. D. Ritchie , et al., “Mitochondrial Damage Contributes to *Pseudomonas aeruginosa* Activation of the Inflammasome and Is Downregulated by Autophagy,” Autophagy 11, no. 1 (2015): 166–182. 25700738 10.4161/15548627.2014.981915 PMC4502769

[37] M. Onishi , K. Yamano , M. Sato , N. Matsuda , and K. Okamoto , “Molecular Mechanisms and Physiological Functions of Mitophagy,” EMBO Journal 40, no. 3 (2021): e104705. 33438778 10.15252/embj.2020104705 PMC7849173

[38] R. A. Williams , T. K. Smith , B. Cull , J. C. Mottram , and G. H. Coombs , “ATG5 is Essential for ATG8‐Dependent Autophagy and Mitochondrial Homeostasis in *Leishmania major* ,” PLoS Pathogens 8, no. 5 (2012): e1002695. 22615560 10.1371/journal.ppat.1002695 PMC3355087

[39] X. Li , J. Wu , X. Sun , et al., “Autophagy Reprograms Alveolar Progenitor Cell Metabolism in Response to Lung Injury,” Stem Cell Reports 14, no. 3 (2020): 420–432. 32059792 10.1016/j.stemcr.2020.01.008 PMC7066233

[40] L. Gui , H. Qian , K. A. Rocco , L. Grecu , and L. E. Niklason , “Efficient Intratracheal Delivery of Airway Epithelial Cells in Mice and Pigs,” American Journal of Physiology Lung Cellular and Molecular Physiology 308, no. 2 (2015): L221–L228. 25416381 10.1152/ajplung.00147.2014 PMC4338941

[41] X. Jia , B. Liu , L. Bao , et al., “Delayed Oseltamivir Plus Sirolimus Treatment Attenuates H1N1 Virus‐Induced Severe Lung Injury Correlated With Repressed NLRP3 Inflammasome Activation and Inflammatory Cell Infiltration,” PLoS Pathogens 14, no. 11 (2018): e1007428. 30422993 10.1371/journal.ppat.1007428 PMC6258564

[42] H. Xian , K. Watari , E. Sanchez‐Lopez , et al., “Oxidized DNA Fragments Exit Mitochondria via mPTP‐ and VDAC‐Dependent Channels to Activate NLRP3 Inflammasome and Interferon Signaling,” Immunity 55, no. 8 (2022): 1370–1385. 35835107 10.1016/j.immuni.2022.06.007 PMC9378606

[43] S. Hanzelmann , R. Castelo , and J. Guinney , “GSVA: Gene Set Variation Analysis for Microarray and RNA‐seq Data,” BMC Bioinformatics [Electronic Resource] 14 (2013): 7. 23323831 10.1186/1471-2105-14-7 PMC3618321

[44] T. Stuart , A. Butler , P. Hoffman , et al., “Comprehensive Integration of Single‐Cell Data,” Cell 177, no. 7 (2019): 1888–1902 e21. 31178118 10.1016/j.cell.2019.05.031 PMC6687398

[45] J. Wang , M. Jiang , A. Xiong , et al., “Integrated Analysis of Single‐Cell and Bulk RNA Sequencing Reveals Pro‐Fibrotic PLA2G7(High) Macrophages in Pulmonary Fibrosis,” Pharmacological Research 182 (2022): 106286. 35662628 10.1016/j.phrs.2022.106286

[46] G. Finak , A. McDavid , M. Yajima , et al., “MAST: A Flexible Statistical Framework for Assessing Transcriptional Changes and Characterizing Heterogeneity in Single‐Cell RNA Sequencing Data,” Genome Biology 16 (2015): 278. 26653891 10.1186/s13059-015-0844-5 PMC4676162

[47] S. Aibar , C. B. Gonzalez‐Blas , T. Moerman , et al., “SCENIC: Single‐Cell Regulatory Network Inference and Clustering,” Nature Methods 14, no. 11 (2017): 1083–1086. 28991892 10.1038/nmeth.4463 PMC5937676

[48] J. Wang , L. Zhang , L. Luo , et al., “Characterizing Cellular Heterogeneity in Fibrotic Hypersensitivity Pneumonitis by Single‐Cell Transcriptional Analysis,” Cell Death Discovery 8, no. 1 (2022): 38. 35091537 10.1038/s41420-022-00831-x PMC8795750

