# Supplementary material for: Epithelial Atg5 Deficiency Intensifies Caspase‐11 Activation, Fueling Extracellular mtDNA Release to Activate cGAS–STING–NLRP3 Axis in Macrophages During Pseudomonas Infection
Source: MedComm (2020). 2025 Jun 15;6(7):e70239. doi: 10.1002/mco2.70239 (PMC12167704; doi:10.1002/mco2.70239)
Supplement: Supplementary file 1 — Supporting Information [file MCO2-6-e70239-s001.pdf]

Epithelial *Atg5* Deficiency Intensifies Caspase-11 Activation, Fueling Extracellular mtDNA Release to Activate cGAS-STING-NLRP3 Axis in Macrophages during *Pseudomonas* Infection

Junyi Wang[1,2,3, #], Lei Zhang[1,2,3, #], Yingying Liu[3,4, #], Yao Liu[2], Anying Xiong[1], Qin Ran[1], Xiang He[1], Vincent Kam Wai Wong[2], Colin Combs[3], Guoping Li [1, *], and Min Wu [4, *]

1 Laboratory of Allergy and Precision Medicine, Department of Respiratory Medicine, Chengdu Institute of Respiratory Health, the Third People's Hospital of Chengdu, Affiliated Hospital of Southwest Jiaotong University, Chengdu 610031, China.

2 State Key Laboratory of Quality Research in Chinese Medicine, Macau University of Science & Technology, Taipa, Macao Special Administrative Region of China 999078, China.

3 Department of Biomedical Sciences, School of Medicine and Health Sciences, University of North Dakota, Grand Forks, ND 58203, USA.

4 Wenzhou Institute, University of Chinese Academy of Sciences, Wenzhou, 325000, Zhejiang, China.

# Junyi Wang, Lei Zhang, and Yingying Liu have contributed equally to this work.

***Correspondence:**

Guoping Li, Laboratory of Allergy and Precision Medicine, Department of Respiratory Medicine, Chengdu Institute of Respiratory Health, the Third People's Hospital of Chengdu, Affiliated Hospital of Southwest Jiaotong University, Chengdu 610031, China.

Email: lzlgp@163.com

Min Wu, Wenzhou Institute, University of Chinese Academy of Sciences, Wenzhou, 325000,

Zhejiang, China.

Email: minwoo2022@126.com

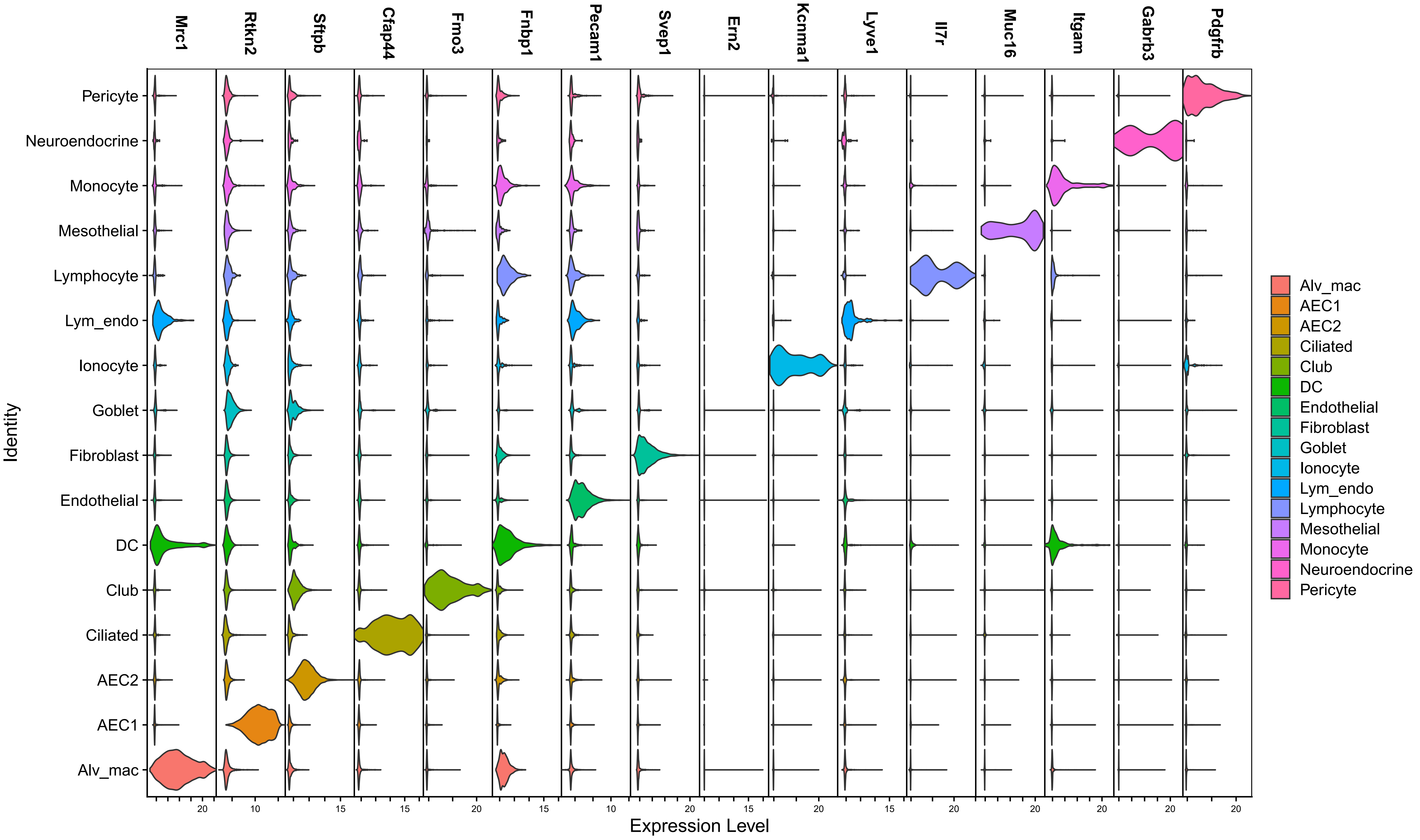
Mrc1
Rtkn2
Sftpb
Cfap44
Fmo3
Fnbp1
Pecam1
Svep1
Ern2
Kcnma1
Lyve1
Il7r
Muc16
Itgam
Gabrb3
Pdgfrb
Pericyte
Neuroendocrine
Monocyte
Mesothelial
Lymphocyte
Lym_endo
Ionocyte
Goblet
Fibroblast
Endothelial
DC
Club
Ciliated
AEC2
AEC1
Alv_mac
Identity
Expression Level
Alv_mac
AEC1
AEC2
Ciliated
Club
DC
Endothelial
Fibroblast
Goblet
Ionocyte
Lym_endo
Lymphocyte
Mesothelial
Monocyte
Neuroendocrine
Pericyte

Figure S1. Stacked violin plot showing the marker gene of each cell type.

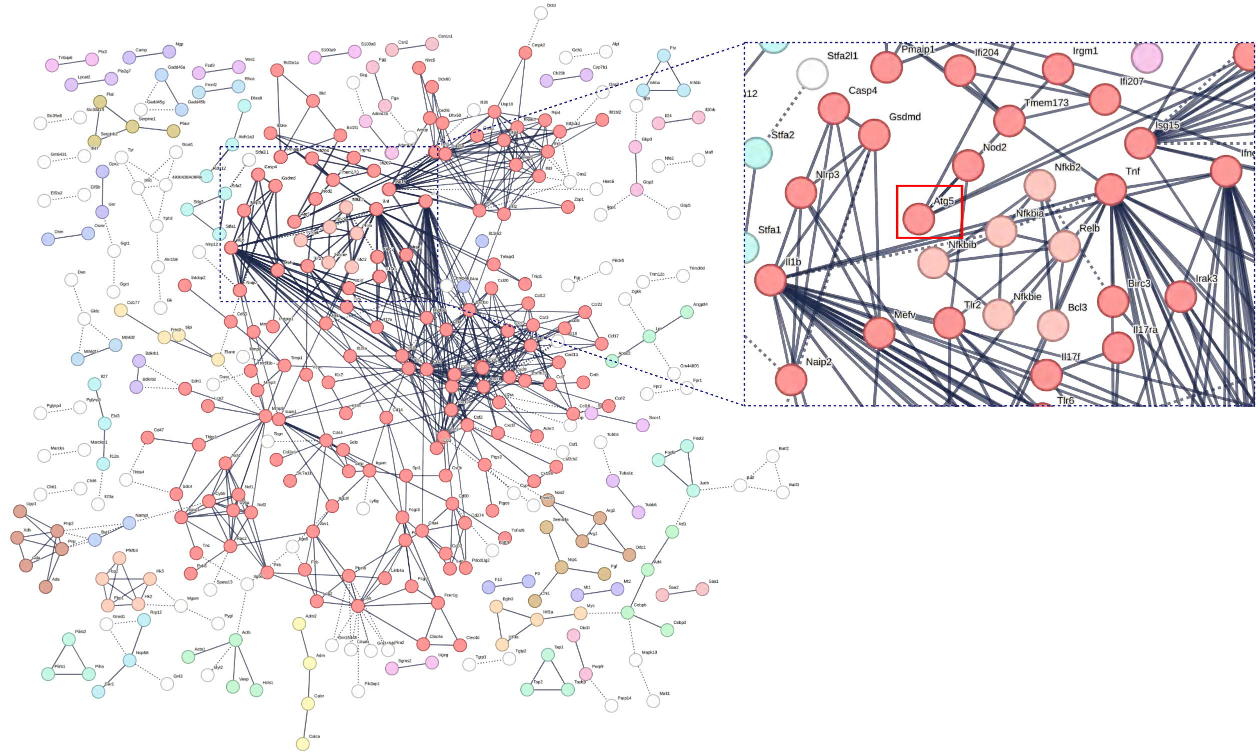
Stfa2l1
Pmaip1
Ifi204
Irgm1
Ifi207
Casp4
Tmem173
Gsdmd
Isg15
Stfa2
Nod2
Nfkb2
Tnf
Nlrp3
Atg5
Nfkbia
Relb
Nfkbib
Stfa1
Il1b
Nfkbie
Birc3
Irak3
Tlr2
Bcl3
Mefv
Il17ra
Il17f
Naip2
Tlr6

Figure S2. Protein-protein interaction (PPI) analysis with differentially expressed genes from the bulk RNA-seq dataset.

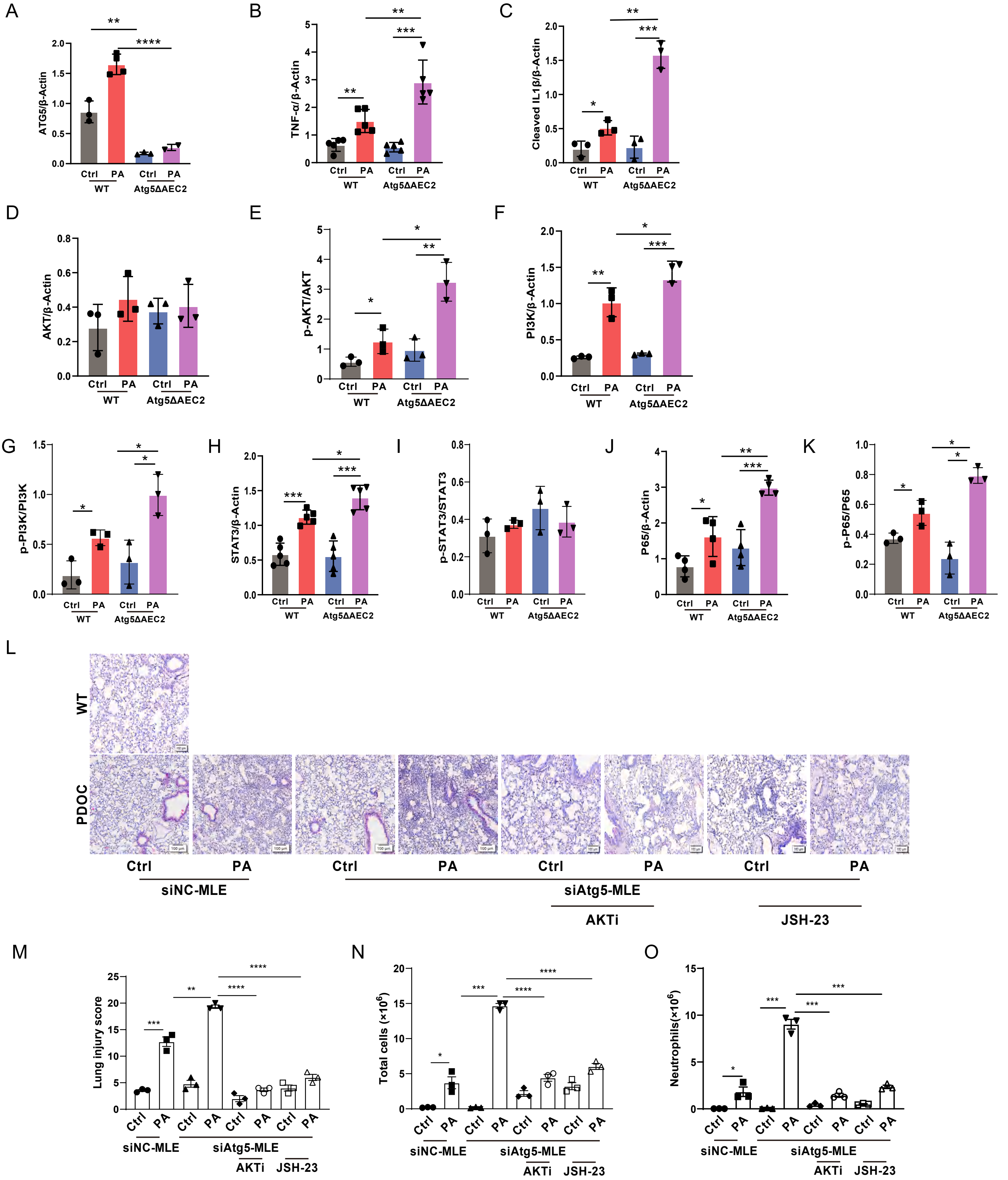
A
ATG5/β-Actin
**
****
Ctrl PA Ctrl PA
WT Atg5ΔAEC2
B
TNF-α/β-Actin
**
***
**
C
Cleaved IL1β/β-Actin
*
**
***
D
AKT/β-Actin
E
p-AKT/AKT
*
**
F
PI3K/β-Actin
**
***
G
p-PI3K/PI3K
H
STAT3/β-Actin
I
p-STAT3/STAT3
J
P65/β-Actin
K
p-P65/P65
L
WT
PDOC
Ctrl PA Ctrl PA Ctrl PA Ctrl PA
siNC-MLE
siAtg5-MLE
AKTi
JSH-23
M
Lung injury score
N
Total cells (×10^6)
O
Neutrophils(×10^6)
Ctrl PA Ctrl PA Ctrl PA Ctrl PA
siNC-MLE siAtg5-MLE
AKTi JSH-23

Figure S3. (A-C) Semiquantitative analysis of ATG5, TNF-α, and IL-1β bands in Figure 2F. Data represent means ± SD of 3 independent experiments, **: $p < 0.01$, ***: $p < 0.001$, ****: $p < 0.0001$ by two-way ANOVA with Bonferroni's post-hoc test. (D-K) Semiquantitative analysis of AKT, PI3K, P65, STAT3, phosphorylated-AKT, phosphorylated-PI3K, phosphorylated-P65, and phosphorylated-STAT3 bands in Figure 2H. Data represent means ± SD of 3 independent experiments, *: $p < 0.05$, **: $p < 0.01$, ***: $p < 0.001$, ****: $p < 0.0001$ by two-way ANOVA with Bonferroni's post-hoc test. WT: wildtype, PA: P. aeruginosa, Ctrl: control, Atg5ΔAEC2: type II alveolar epithelial cell-specific Atg5 conditional knockout. (L) Representative histological images and injury scores (M) of murine lungs via H&E staining (Scale bar represents 100 µm). Data represent means ± SD (n= 3), ****: $p < 0.0001$ by two-way ANOVA with Bonferroni's post-hoc test. (N-O) The number of total cells (N) and neutrophils (O) in BALF. Data represent means ± SD (n= 3), *: $p < 0.05$, **: $p < 0.01$, ***: $p < 0.001$, ****: $p < 0.0001$ by one-way ANOVA with Bonferroni's post-hoc test. Ctrl: control, PDOC: intratracheally administered of polidocanol, PA: P. aeruginosa, SiNC-MLE: intratracheal injection of MLE-12 cells transfected with control siRNA, siAtg5-MLE: intratracheal injection of MLE-12 cells transfected with Atg5 siRNA, AKTi: treated with the AKTi ½, JSH-23: treated with the JSH-23.

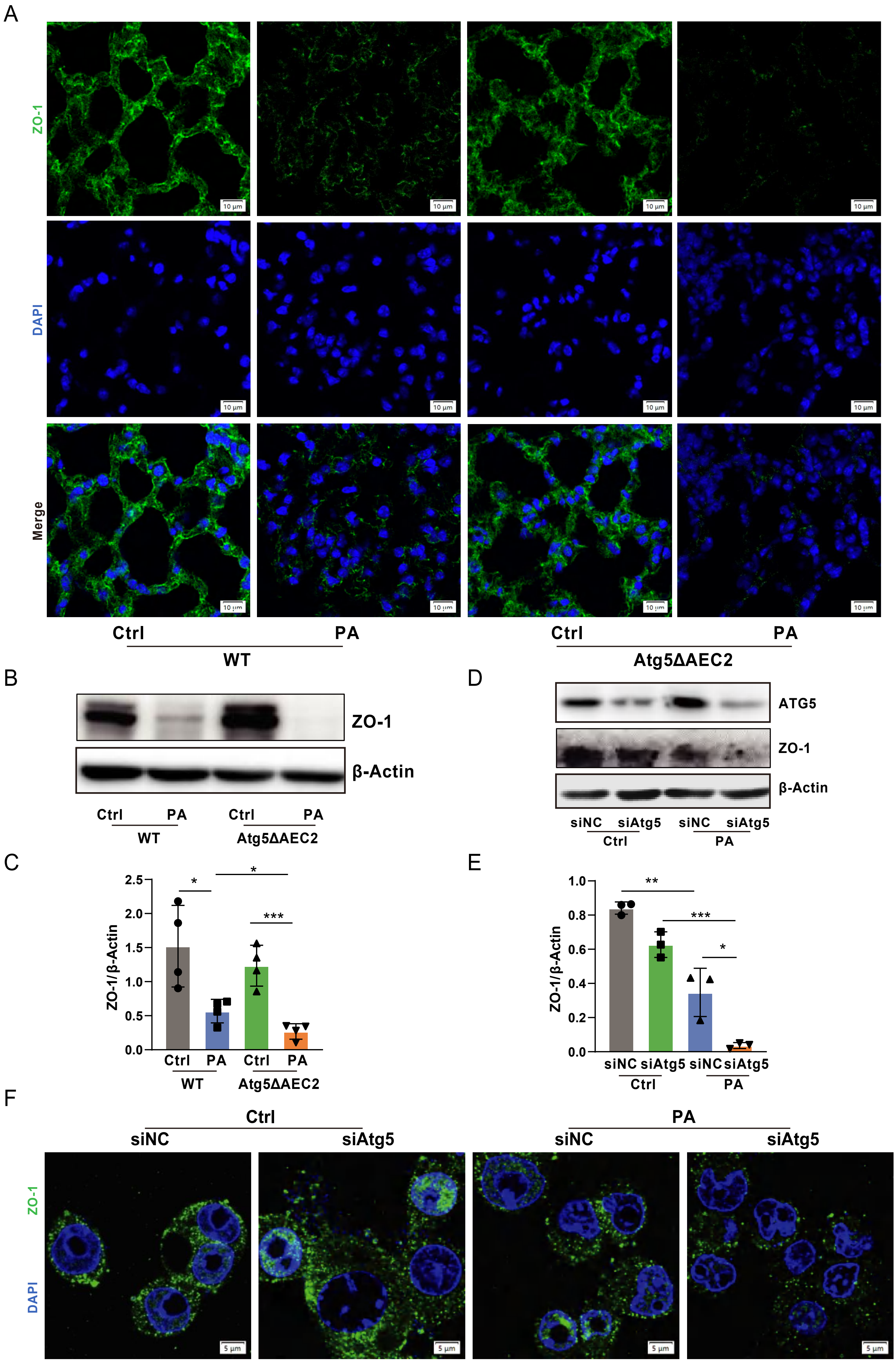
A
ZO-1
DAPI
Merge
10 μm
Ctrl
PA
WT
Ctrl
PA
Atg5ΔAEC2
B
ZO-1
β-Actin
Ctrl
PA
WT
Ctrl
PA
Atg5ΔAEC2
C
ZO-1/β-Actin
2.5
2.0
1.5
1.0
0.5
0.0
*
*
***
Ctrl
PA
WT
Ctrl
PA
Atg5ΔAEC2
D
ATG5
ZO-1
β-Actin
siNC
siAtg5
Ctrl
siNC
siAtg5
PA
E
ZO-1/β-Actin
1.0
0.8
0.6
0.4
0.2
0.0
**
***
*
siNC
siAtg5
siNC
siAtg5
Ctrl
PA
F
Ctrl
siNC
siAtg5
PA
siNC
siAtg5
ZO-1
DAPI
5 μm

Figure S4. (A) ZO-1 expression was analyzed by immunofluorescence in murine lungs (Scale bar represents 5 μm). (B) The expression levels of ZO-1 in mouse lungs determined by western blotting. (C) Semiquantitative analysis of (B). Data represent means ± SD of 4 independent experiments, *: $p < 0.05$, ***: $p < 0.001$ by two-way ANOVA with Bonferroni's post-hoc test. (D) The expression levels of ZO-1 in MLE-12 cells determined by western blotting. (E) semiquantitative analysis of (D). Data represent means ± SD of 3 independent experiments, *: $p < 0.05$, **: $p < 0.01$, ***: $p < 0.001$ by two-way ANOVA with Bonferroni's post-hoc test. (F) ZO-1 expression was analyzed by immunofluorescence in MLE-12 cells (Scale bar represents 5 μm). WT: wildtype, PA: P. aeruginosa, Ctrl: control, Atg5ΔAEC2: type II alveolar epithelial cell-specific Atg5 conditional knockout, siNC: control siRNA, siAtg5: Atg5 siRNA.

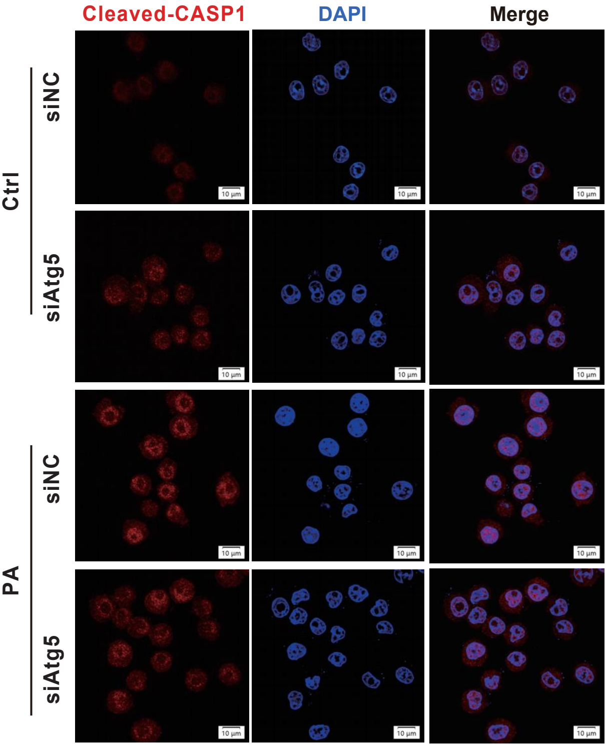

Cleaved-CASP1
DAPI
Merge
Ctrl
siNC
siAtg5
PA
siNC
siAtg5
10 μm

Figure S5. The expression of cleaved CASP1 was analyzed by immunofluorescence in MLE-12 cells (Scale bar represents 10 μm). Ctrl: control, PA: P. aeruginosa, siNC: control siRNA, siAtg5: Atg5 siRNA.

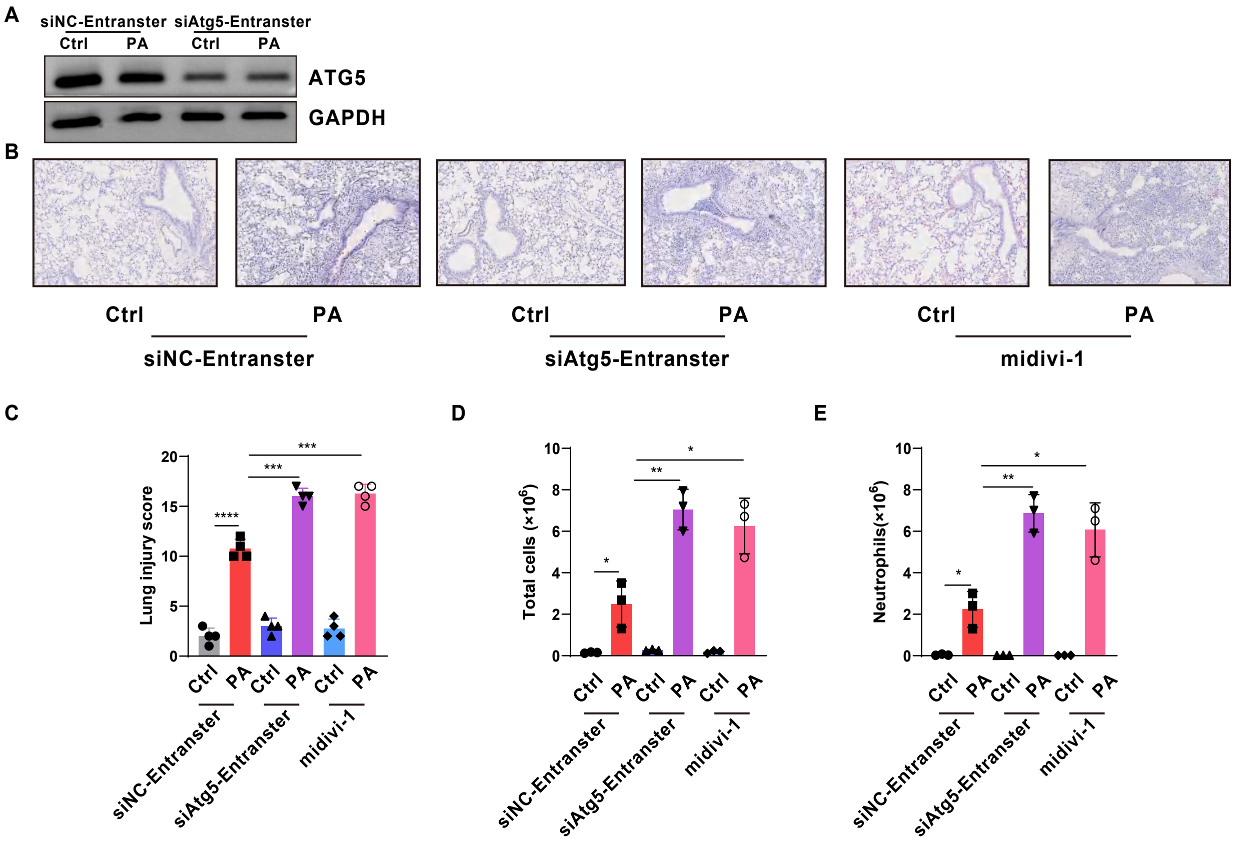

A
siNC-Entranster
siAtg5-Entranster
Ctrl
PA
ATG5
GAPDH
B
Ctrl
PA
siNC-Entranster
siAtg5-Entranster
midivi-1
C
Lung injury score
****
***
D
Total cells (×10^6)
E
Neutrophils(×10^6)

Figure S6. (A) The expression levels of ATG5 in mouse lungs determined by western blotting. (B) Representative histological images and injury scores (C) of murine lungs via H&E staining (Scale bar represents 100 μm). Data represent means ± SD (n= 4), *: $p < 0.05$, **: $p < 0.01$, ***: $p < 0.001$, ****: $p < 0.0001$ by two-way ANOVA with Bonferroni's post-hoc test. (D-E) The number of total cells (D) and neutrophils (E) in BALF. Data represent means ± SD (n= 3), *: $p < 0.05$, **: $p < 0.01$, ***: $p < 0.001$, ****: $p < 0.0001$ by one-way ANOVA with Bonferroni's post-hoc test. Ctrl: control, PA: P. aeruginosa, siNC-Entranster: intranasal transfection of control siRNA with Entranster™-in vivo reagent, siAtg5-Entranster: intranasal transfection of Atg5 siRNA with Entranster™-in vivo reagent, midivi-1: treated with the Mdivi-1.

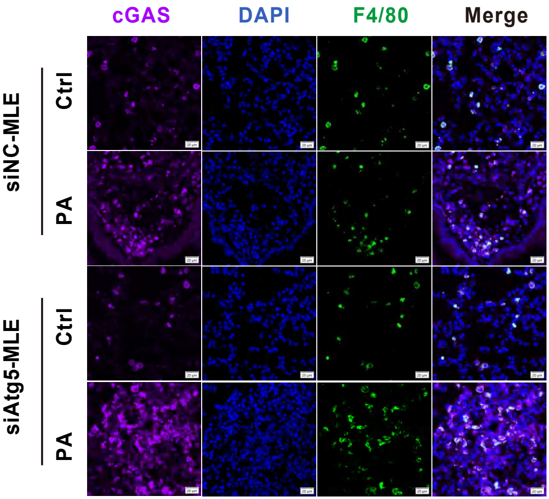
cGAS
DAPI
F4/80
Merge
siNC-MLE
Ctrl
PA
siAtg5-MLE
Ctrl
PA

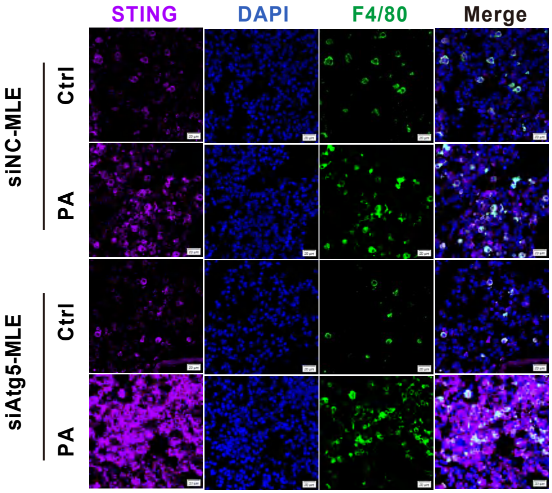
STING
DAPI
F4/80
Merge
siNC-MLE
Ctrl
PA
siAtg5-MLE
Ctrl
PA

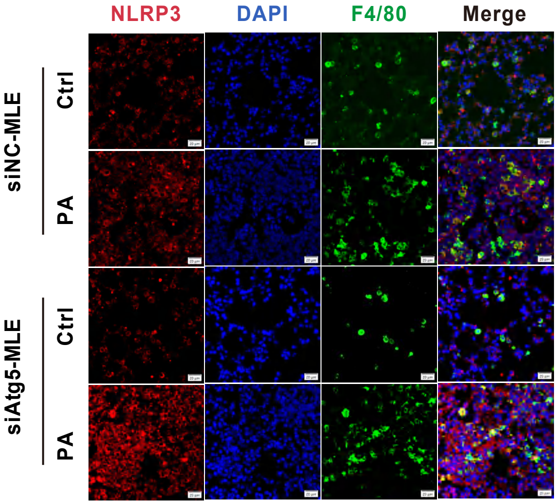
NLRP3
DAPI
F4/80
Merge
siNC-MLE
Ctrl
PA
siAtg5-MLE
Ctrl
PA

Figure S7. cGAS, STING, and NLRP3 expression was analyzed by immunofluorescence in macrophages (stained with F4/80) of murine lungs (Scale bar represents 20 μm). Ctrl: control, PA: P. aeruginosa, SiNC-MLE: intratracheal injection of MLE-12 cells transfected with control siRNA, siAtg5-MLE: intratracheal injection of MLE-12 cells transfected with Atg5 siRNA

Table S1：Autophagy related gene signatures

Positive regulator of Autophagy	Negative regulator of Autophagy genes	Negative Regulator of Autophagy	ATG9 and its cycling system	PI3K complex-class 1	ATG12 Ubiquitin-like conjugation systems
Abl1	Atf5	3110043O21Rik	Atg2a	Pik3c2a	Atg10
Abl2	Bdnf	Capn1	Atg2b	Pik3c2b	Atg12
Bag3	Jun	Capn2	Atg9a	Pik3c2g	Atg16l1
Cast	Junb	Capns1	Atg9b	Pik3ca	Atg16l2
Dapk1	Keap1	Cisd2	Wdr45	Pik3cb	Atg5
Dapk2	Nr1h4	Cul3	Wdr45b	Pik3cd	Atg7
Dapk3	Stat1	Cul4a	Wipi1	Pik3cg	
Dram1	Zkscan3	Cul4b	Wipi2	Pik3r1	
Dynll1		Cul5		Pik3r2	
Ei24		Cybb		Pik3r3	
Eva1a		Fez1		Pik3r4	
Hdac1		Golga2		Pik3r5	
Hdac10		Herc1		Pik3r6	
Hdac2		Hspa1a			
Irgm2		1700021K19Rik			
5330417C22Rik		Mapk14			
Mapk8		Mst1			
Mcoln3		Nedd4l			
Mtdh		Ppp2r2a			
Myo6		Stk39			
Nox4		Tmem173			
Pcm1		Ucp2			
Pdcd6ip		Wnk1			
Picalm					
Pim2					
Pxn					
Rab18					
Rab3gap1					
Scoc					
Smurf1					
Snap29					
Stub1					
Tbk1					
Tgm2					
Tm9sf1					
Tmem59					
Tmem74					
Wac					
Zfyve1					

Rab proteins involved in autophagosome formation	Positive regulator of Autophagy genes	pyroptosis	mTOR regulators	mTORC2 substrates	mTOR complexes 1 and 2	mTORC1 substrates	RAS family	Autophahe Formation
Rab11a	Atf2	Aim2	Atp6ap1	Akt1	Akt1s1	Akt1s1	Hras	Ambra1
Rab11b	Atf3	Bak1	Atp6ap2	Hdac4	Deptor	Ambra1	Kras	Atg3
Rab1	Atf4	Bax	Atp6v0a1	Hdac5	Mapkap1	Atg13	Mras	Atg4A
Rab1b	Atf6	Casp1	Atp6v0a2	Igf2bp1	Mlst8	Dap	Nras	Atg4B
Rab23	Cebpb	Casp3	Atp6v0a4	Prkca	Mtor	Eef2k	Rras	Atg4C
Rab24	Creb1	Casp4	Atp6v0b	Sgk1	Prr5	Eif4ebp1		Atg4D
Rab25	Crtc2	Casp6	Atp6v0c		Prr5l	Esr1		Atg5
Rab32	Ddit3	Casp8	Atp6v0d1		Rictor	Grb10		Atg7
Rab33b	E2f1	Casp9	Atp6v0e		Rptor	Hif1a		Atg9A
Rab4a	Egr1	Chmp2a	Atp6v0e2		Telo2	Larp1		Atg9B
Rab4b	Eif2a	Chmp2b	Atp6v1a		Tti1	Lpin1		Atg10
Rab5a	Epas1	Chmp3	Atp6v1b1		Tti2	Maf1		Atg12
Rab5b	Esr2	Chmp4b	Atp6v1b2			Mitf		Atg16L1
Rab5c	Fos	Chmp4c	Atp6v1c1			Rps6ka1		Atg16L2
Rab7	Foxo1	Chmp6	Atp6v1c2			Rps6kb1		Arsa
Rab8a	Foxo3	Chmp7	Atp6v1d			Rps6kb2		Becn1
Rab8b	Foxo4	Cycs	Atp6v1e1			Stat3		Becn1L1
Rab9	Foxo6	Elane	Atp6v1e2			Tfe3		Dram1
	Hey2	Gpx4	Atp6v1f			Tfeb		Gabarap
	Hif1a	Gsdma	Atp6v1g1			Ulk1		Gabarapl1
	Hsf1	Gsdmc4	Atp6v1g2			Yy1		Gabarapl2
	Irf1	Gsdmc	Atp6v1g3					Hgs
	Lmx1a	Gsdmc3	Atp6v1h					Irgm
	Lmx1b	Gsdmc2	Btrc					Lamp1
	Mapk1	Gsdmd	BC048403					Lamp2
	Mapk3	Il18	Depdc5					Lamp3
	Mef2a	Il1a	Flcn					Map1Lc3A
	Mitf	Il1b	Fnip1					Map1Lc3B
	Myc	Il6	Fnip2					Map1Lc3B2
	Nacc1	Irf1	Itfg2					Map1Lc3C
	Nfe2l1	Irf2	C330027C09Rik					Pik3C3
	Nfe2l2	Nlrc4	Kptn					Pik3R4
	Nfkb1	Nlrp1b	Lamtor1					Rubicon
	Nrf1	Nlrp2	Lamtor2					Rab24
	Ppara	Nlrp3	Lamtor3					Sec16A
	Pparg	Nlrp6	Lamtor4					Sec16B
	Ppargc1a	Nod1	Lamtor5					Sec23A
	Ppargc1b	Nod2	Lgals8					Sec23B
	Rara	Plcg1	Mios					Sec24A
	Rela	Prkaca	Npc1					Sec24B
	Relb	Pycard	Npc2					Sec24C
	Rfx1	Scaf11	Nprl2					Sec24D
	Sirt1	Tirap	Nprl3					Tmem74
	Sirt2	Tnf	Ppp2ca					Ulk1
	Sirt3	Trp53	Rheb					Uvrag
	Sirt4	Trp63	Rraga					Ulk2
	Sirt5		Rragb					Ulk3
	Sirt6		Rragc					Ulk4
	Sirt7		Rragd					Wipi1
	Sox2		Sec13					Wdr45
	Sp1		Seh1l					Wdr45B
	Stat3		Sesn1					Wipi2
	Tfe3		Sesn2					
	Tfeb		Sesn3					
	Tfec		Slc38a9					
	Tnfaip3		Szt2					
	Trp53		Tcirg1					
	Xbp1		Tsc1					
	Yy1		Tsc2					
	Zeb1		Wdr24					
			Wdr59					
